# Habitat type and host grazing regimen influence the soil microbial diversity and communities within potential biting midge larval habitats

**DOI:** 10.1186/s40793-022-00456-8

**Published:** 2023-01-19

**Authors:** Saraswoti Neupane, Travis Davis, Dana Nayduch, Bethany L. McGregor

**Affiliations:** 1 Department of Entomology, Kansas State University, Manhattan, KS 66506 USA; 2 Arthropod-Borne Animal Diseases Research Unit, USDA-ARS, Center for Grain and Animal Health Research, Manhattan, KS 66502 USA

**Keywords:** Potential midge larval habitat, Soil, rRNA gene, Bacteria, Protists, Fungi, Metazoa community, Diversity

## Abstract

**Background:**

Biting midges (*Culicoides* spp.) are important vectors of diverse microbes such as viruses, protozoa, and nematodes that cause diseases in wild and domestic animals. However, little is known about the role of microbial communities in midge larval habitat utilization in the wild. In this study, we characterized microbial communities (bacterial, protistan, fungal and metazoan) in soils from disturbed (bison and cattle grazed) and undisturbed (non-grazed) pond and spring potential midge larval habitats. We evaluated the influence of habitat and grazing disturbance and their interaction on microbial communities, diversity, presence of midges, and soil properties.

**Results:**

Bacterial, protistan, fungal and metazoan community compositions were significantly influenced by habitat and grazing type. Irrespective of habitat and grazing type, soil communities were dominated by phyla Acidobacteria, Actinobacteria, Bacteroidetes, Chloroflexi, Firmicutes, Proteobacteria (Bacteria); Apicomplexa, Cercozoa, Ciliophora, Ochrophyta (Protists); Chytridiomycota, Cryptomycota (Fungi) and Nematoda, Arthropoda (Metazoa). The relative abundance of Acidobacteria, Actinobacteria, Bacteroidetes, Chloroflexi, Firmicutes, Proteobacteria, Verrucomicrobia (Bacteria); Apicomplexa, Lobosa (Protists); Ascomycota, Blastomycotina, Cryptomycota (Fungi); and Platyhelminthes (Metazoa) were significantly affected by grazing type. Of note, midge prevalence was higher in grazed sites (67–100%) than non-grazed (25%). Presence of midges in the soil was negatively correlated with bacterial, protistan, fungal and metazoan beta diversities and metazoan species richness but positively correlated with protistan and fungal species richness. Moreover, total carbon (TC), nitrogen (TN) and organic matter (OM) were negatively correlated with the presence of midges and relative abundances of unclassified Solirubrobacterales (Bacteria) and Chlamydomonadales (Protists) but positively with Proteobacteria and unclassified Burkholderiales (Bacteria).

**Conclusions:**

Habitat and grazing type shaped the soil bacterial, protistan, fungal and metazoan communities, their compositions and diversities, as well as presence of midges. Soil properties (TN, TC, OM) also influenced soil microbial communities, diversities and the presence of midges. Prevalence of midges mainly in grazed sites indicates that midges prefer to breed and shelter in a habitat with abundant hosts, probably due to greater accessibility of food (blood meals). These results provide a first glimpse into the microbial communities, soil properties and prevalence of midges in suspected midge larval habitats at a protected natural prairie site.

**Supplementary Information:**

The online version contains supplementary material available at 10.1186/s40793-022-00456-8.

## Introduction

Biting midges (*Culicoides* spp.) are small, blood-feeding flies that serve as important vectors of diverse microbes such as viruses, protozoans, and nematodes that cause diseases in wild and domestic animals [1]. Several of these *Culicoides-*borne pathogens, especially the viruses, can cause morbidity and mortality to livestock and significant economic impacts to farmers and ranchers. For example, an outbreak of the *Culicoides-*borne bluetongue virus serotype 8 in Germany caused between 157 and 203 million Euros in direct and indirect costs to dairy cattle, beef cattle, and sheep industries [2]. Another *Culicoides*-borne pathogen in the Americas, vesicular stomatitis virus (VSV), can cause not only significant direct economic impacts in animal injury and lost productivity [3–6], but also losses from movement restrictions due to VSV being a reportable disease in the United States. Understanding the biology and ecology of biting midges is an essential step towards improving and implementing effective control strategies to mitigate transmission risk and potential economic impacts of midge-transmitted diseases.

The larval and pupal stages of most *Culicoides* species require semi-aquatic moist soil habitats. Typically, these stages are found at the soil–water interface of diverse waterbodies, including ponds, streams, springs, and marshes [7]. Many species also are associated with habitats that are organically enriched, especially due to input of animal waste [8, 9]. While there have been some studies correlating the relationship between soil chemistry and the presence of *Culicoides* species [9–11], these studies are lacking for the Great Plains region of the United States. Additionally, little is currently known about the role of microbial communities on the presence, abundance, and emergence of midges from their natural habitats.

Microbial communities play a crucial role in survival, development, and fitness of dipteran pests and vectors. For instance, larvae of the higher dipteran pests *Musca domestica* L., and *Stomoxys calcitrans* L. failed to develop and survive without being provisioned live microbes in their diet, indicating that the microbes and/or microbial metabolites provided essential larval nutrition [12, 13]. Further, microbial communities of dairy cattle manure (a natural larval habitat for house flies) were significantly altered by *M. domestica* larval grazing, indicating a possible role of microbial communities in larval survival and development [14]. Similarly, *Culicoides stellifer* also required microbial communities including bacteria and nematodes in their diet for survival and development [15]. Indirect evidence of bacterial requirements for *Culicoides variipennis* was shown in a study that demonstrated that water samples from both natural habitats and laboratory rearing pans shared a number of bacterial taxa, as did field-caught and lab-reared pupae and adults [16]. However, no further efforts have been made to explore the microbial communities in natural breeding sites of *Culicoides* and the correlation between habitats and the presence of *Culicoides*. Here we simultaneously characterized bacterial, protistan, fungal and small soil invertebrate communities in soils from disturbed (bison and cattle grazed) and undisturbed (non-grazed) ponds and springs (putative midge larval habitats) and evaluated the influence of habitat and grazing disturbances on these microbial communities, their diversities and midge presence.

## Materials and methods

### Experimental fields, soil sample collection and physicochemical analysis

Soil collections were conducted at the Konza Prairie Biological Station (KPBS), which is located just south of Manhattan, Kansas, USA. This site is represented by hilly tallgrass prairie over limestone embedded soils interspersed with natural water sources such as ponds, streams, and springs. The site contains separate bison-grazed and cattle-grazed subsections as well as a large area where no formal grazing is conducted (non-grazed). The non-grazed area contains a human walking trail and all three grazing regimens possess abundant white-tailed deer.

Six sampling sites (suspected midge larval habitats) were selected, representing two typical larval habitats (pond and spring) located within the three grazing regimens (bison-grazed, cattle-grazed, and non-grazed). Soil samples were collected from September to December 2020 from each site. A composite sample of approximately 500 g soil was collected from 0 to 5 cm depth and 10 random locations within a sampling site using a small garden trowel. Sampling was prioritized near the soil–water interface where midge larvae are typically abundant. Soil samples were collected into plastic bags and brought back to the laboratory for further analysis. Four hundred grams of the collected soil sample was used to assess midge presence and abundance via emergence assays (described below) and an aliquot of ~ 50 g soil sample was stored at – 80 °C for subsequent molecular and physicochemical analysis. For physicochemical analysis, ~ 30 g of soil from each sample was sent to the Soil Testing Lab, Department of Agronomy, Kansas State University (https://www.agronomy.k-state.edu/services/soiltesting/) and analyzed for total carbon (TC), total nitrogen (TN), and organic matter (OM).

### Midge emergence assays

Due to challenges in isolating and identifying midges in the larval stage, emergence assays were used to evaluate the presence and abundance of midge larvae in soil collections. Two hundred grams of collected soil was placed onto large petri dishes (100 mm × 26 mm; ThermoFisher Scientific, Waltham, MA, USA) in duplicate, and incubated in an environmental chamber (Model 136VL, Percival Scientific Inc, Perry, IA, USA) at 27 °C with a pan of deionized water to maintain humidity ~ 80% at a 12:12 (light:dark) photoperiod for up to 6-weeks post collection to encourage the completion of development and emergence of adult *Culicoides* midges. Three times per week, the petri dishes were removed from the environmental chamber, placed into 9.5 L plastic bags, and opened to allow recently emerged *Culicoides* to fly into the bag. Midges that flew into the bag were then collected by battery-powered aspirator (2809B InsectaVac Aspirator, Bioquip, Inc., USA), identified to species using morphological keys and identification aids [7, 17, 18], and placed into 1.5 ml microcentrifuge tubes with 95% ethanol.

### DNA extraction, library preparation, sequencing and analysis

Quick-DNA Fecal/Soil Microbe Kits (Zymo Research, Irvine, CA, USA) were utilized to extract soil genomic DNA. DNA was extracted from 0.25 g soil as described in the manufacturer’s protocol. Similarly, for positive and negative controls, DNA was extracted from a laboratory culture of bacterium (*Staphylococcus aureus*) and fungus (*Davidiella* sp.), and nuclease free water, respectively. These control DNA samples were further used as controls for library preparation, sequencing, and sequence analysis. Once extracted, DNA was quantified using the Qubit™ dsDNA HS kit (Thermo Fisher Scientific Inc., Waltham, MA, USA) in a Qubit 4 Fluorometer (Thermo Fisher Scientific Inc., Waltham, MA, USA) and stored at – 20 °C until further analysis.

Amplicon sequencing of the bacterial V3-V4 region of the 16S rRNA gene and the eukaryal V4 region of the 18S rRNA gene was performed using the MiSeq Illumina system to characterize prokaryotic (bacterial) and eukaryotic (protists, fungi and small soil animals (metazoa)) communities, respectively, in soil collected from potential midge larval habitats. Primer pairs for bacterial communities were 341F and 806R [19], and for eukaryal communities were TAReuk454F and TAReukREV3 [20]. Library preparation and sequencing were performed at the Genome Sequencing Core, University of Kansas, Lawrence, Kansas using the 16S metagenome protocol from Illumina (https://tinyurl.com/ybxgxsqm). Sequencing was performed using MiSeq reagent kit v3 (Illumina Inc., USA) for paired-end 2 × 300 bp.

Both bacterial (16S rRNA) and eukaryal (18S rRNA) raw amplicon sequence data were analyzed in Mothur (version 1.45.2, [21]) as described previously [14]. Briefly, primers and low-quality sequence reads were removed, and paired end reads were assembled. Assembled sequence reads were aligned to the SILVA reference sequence alignment database [22] and unaligned sequences were removed. Chimeric sequences were identified using VSEARCH (version 2.16.3, [23]) and were removed. High-quality, non-chimeric, unique sequence reads were clustered into operational taxonomic units (OTUs) if sequences were 97% similar. Consensus taxonomy of representative sequences of OTUs were determined using the Naïve Bayesian Classification method [24] and RDP reference database for bacteria [25] and protist ribosomal reference database (PR^2^) for eukaryotes [26] that includes protists, fungi and metazoa. For bacterial communities, OTUs classified as archaea, mitochondria, eukaryota and unknown were removed. Further, erroneous (OTUs present in negative control, and positive control sample that were classified other than *Staphylococcus aureus*) and low abundant (frequency < 2, and < 0.00001% of total abundance) OTUs were removed. Subsequently, obtained bacterial OTU frequency data was summarized to bacterial phylum and genus levels and was used for subsequent statistical analyses.

For eukaryal communities, low abundance (frequency < 2, and < 0.00001% of total abundance) and erroneous OTUs (OTUs present in negative control, and positive control sample that were classified other than *Davidiella* sp.) were removed. From the final eukaryal OTU table, protistan, metazoan and fungal OTU tables were prepared. For the protistan OTU frequency table, OTUs classified as unknown, metazoan, fungi, unknown archaeplastida, streptophyta, unknown eukaryota, and unknown opisthokonta were removed. The protistan OTU dataset was further summarized to subkingdom, phylum, class and genus level. Further, protists communities were assigned to functional groups (trophic groups) as described in [27–29]. Similarly, metazoan and fungal OTU tables were prepared by selecting OTUs classified as metazoa and fungi, respectively. The OTU tables were summarized to phylum, class, and genus level for each domain and were subsequently used for statistical analyses.

All statistical analyses were performed in the R statistical program (version 3.6.2, [30]). For each microbial community (bacteria, protists, fungi, and metazoa), alpha diversity indices (species richness and Shannon diversity index (*H’*)), Pielou’s evenness and beta diversity were calculated using respective OTU data in the ‘Vegan’ package (version 2.5–7, [31]). The correlation between bacterial, protistan, fungal, and metazoan diversities as well as abundances of major phyla or taxa at their lowest taxonomic resolution (genus), soil properties (TN, TC and OM) and the presence of *Culicoides* spp. were determined using Pearson correlation coefficient. Also, the effects of grazing type, habitat type and their interaction on the presence of midges, soil properties and microbial diversity was assessed by a general or generalized linear model where response variables were presence of midges, soil properties, microbial diversity or abundance of microbial taxa. The predictor variables were habitat, grazing types and their interaction. Post hoc comparison of least square mean was determined between habitat types, across grazing types and/or between habitat types. A principal coordinate analysis (PCoA) was used to determine the microbial (bacterial, protistan, fungal and metazoan) community composition in each sample. Bray–Curtis dissimilarity index or Euclidean matrix for each microbial OTU was calculated and the first two PCoA axes were plotted to visualize community composition. In order to examine effect of habitat, grazing type and presence of midges on microbial community composition, a permutational multivariate analysis of variance (Adonis) was used. All statistical tests with *P*-value < 0.05 were considered statistically significant.

## Results

The prevalence of biting midges (irrespective of midge species) in soil collected from disturbed (bison and cattle grazed) and undisturbed (non-grazed) natural habitats varied across sampling sites. Grazing type significantly influenced the presence of midges (χ^2^ = 13.14, df = 2, *P* = 0.0014). Midge prevalence was 100% in both habitats (pond and spring) of cattle-grazed sites and in the pond habitat of bison-grazed sites. The spring habitats in the bison-grazed site had only 66.66% midge prevalence. Surprisingly, midge prevalence was 25% in soil samples from both habitats (pond and spring) of non-grazed sites. Further, habitat type had no significant effect on the presence of midges (prevalence: pond, 72.73%; spring, 63.64%).

### Effects of habitat and grazing on soil properties

**Fig. 1:**
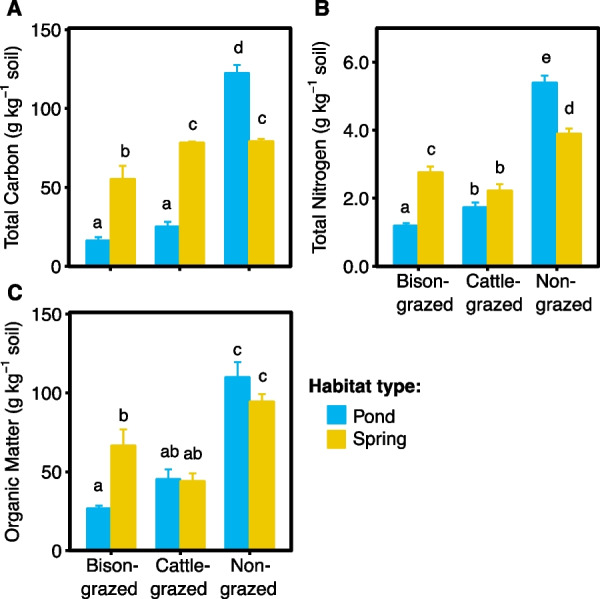
Soil properties of potential midge habitats. Mean **A** Total Carbon, **B** Total Nitrogen, **C** Organic matter in disturbed (bison- and cattle-grazed) and undisturbed (non-grazed) pond and spring habitats. The error bars are standard errors of the means. The different letters on top indicate the significant differences between habitat type and grazing type (*P* ≤ 0.05)

Irrespective of sampling sites, TC in potential midge habitat soil comprised 11.20–134.82 g kg^−1^ dry soil. Grazing type (F_(2,15)_ = 161.34, *P* < 0.0001), habitat type (F_(1,15)_ = 24.14, *P* = 0.0002), and their interaction (F_(2,15)_ = 81.66, *P* < 0.0001) significantly influenced the soil TC. Also, TC was significantly higher in soil from non-grazed habitats compared to bison and cattle grazed habitats for both pond and spring sites (Fig. 1A). Total nitrogen in soil ranged from 1.0 to 5.9 g kg^−1^ dry soil. Grazing type (F_(2,15)_ = 195.49, *P* < 0.0001) and the interaction of grazing and habitat types (F_(2,15)_ = 43.82, *P* < 0.0001) significantly affected the TN content of the soil. Soil from the non-grazed pond had significantly higher TN content compared to the non-grazed spring (t = 6.38, df = 15, *P* = 0.0001), cattle-grazed pond (t = 8.62, df = 15, *P* < 0.0001), cattle-grazed spring (t = 14.59, df = 15, *P* < 0.0001), bison-grazed pond (t = 11.49, df = 15, *P* < 0.0001) and bison-grazed spring (t = 4.51, df = 15, *P* = 0.0045, Fig. 1B). The range of soil organic matter comprised 23.0–138.0 g kg^−1^ dry soil. Grazing type (F_(2,15)_ = 50.70, *P* < 0.0001) and the interaction of grazing and habitat types (F_(2,15)_ = 8.76, *P* = 0.0030) significantly affected the soil organic matter. Similar to TC and TN, soil OM was significantly higher in both pond and spring habitats of non-grazed sites compared to ponds and springs of grazed sites (Fig. 1C).

### Bacterial diversity and communities

**Fig. 2:**
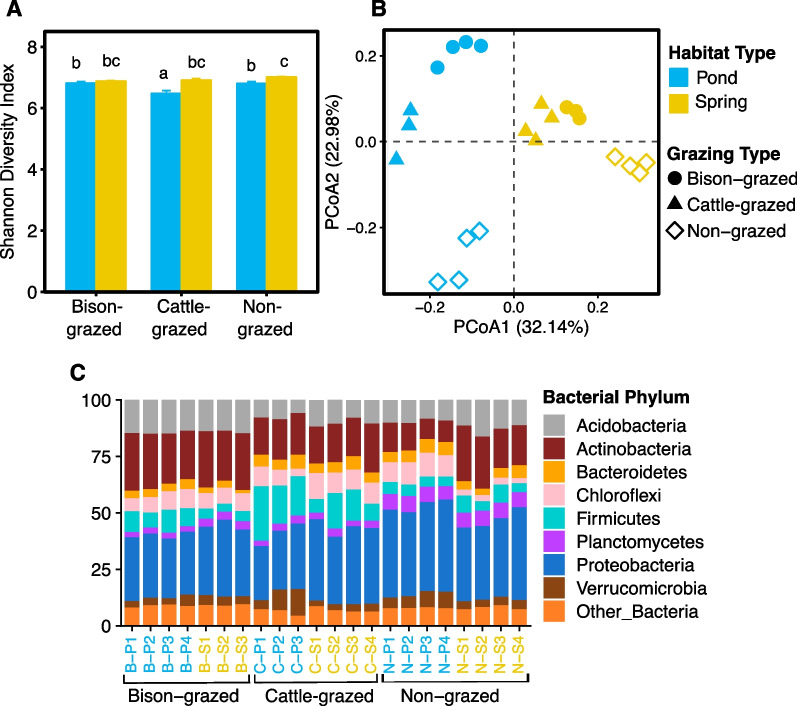
Bacterial diversity and community composition of potential midge habitat soil. **A** Shannon diversity index in disturbed (bison- and cattle-grazed) and undisturbed (non-grazed) pond and spring habitats. The error bars are standard errors of means. The different letters indicate significant differences between sampling sites (*P* ≤ 0.05). **B** Bacterial community composition of each sample. The first and second axes of Principal Co-ordinates Analysis depicts Bray–Curtis distances between samples. **C** Bacterial community composition (phyla). Relative abundance for each sample is shown, and sample names are color coded based on habitat types on the horizontal axis (pond = blue and spring = yellow)

Bacterial 16S rRNA amplicon sequencing comprised 1,174,780 high quality sequence reads that were clustered into 6,508 OTUs. Species richness (observed OTUs) ranged from 2,089 to 2,951 OTUs per sample. Habitat and grazing types and their interaction had no significant effects on bacterial species richness. However, bacterial Shannon diversity index was significantly affected by habitat (F_(2,16)_ = 32.65, *P* < 0.0001) and grazing (F_(2,16)_ = 6.71, *P* = 0.0076) types and their interaction (F_(2,16)_ = 6.31, *P* = 0.0095). Shannon diversity index was significantly lower in the cattle-grazed pond site compared to other sites (Fig. 2A, Additional file 2: Table S1). Bacterial Pielou’s evenness was significantly affected by grazing (F_(2,16)_ = 8.39, *P* = 0.0032), habitat (F_(1,16)_ = 44.07, *P* < 0.0001) types, and their interaction (F_(2,16)_ = 4.96, *P* = 0.0211). Mean evenness was significantly higher in spring (t = 6.69, *P* < 0.0001) than pond habitats. Also, evenness was significantly higher in bison-grazed (t = 4.63, *P* = 0.0008) and non-grazed (t = 3.86, *P* = 0.004) than cattle-grazed sites (Additional file 2: Table S1). Moreover, bacterial β-diversity was significantly influenced by grazing (F_(2,16)_ = 3.98, *P* = 0.0398) and habitat (F_(2,16)_ = 8.66, *P* = 0.0096) types. β-diversity was significantly higher in pond (t = 3.06, *P* = 0.007) compared to spring habitats while non-grazed sites comprised non-significantly higher β-diversity compared to bison- (t = 2.51, *P* = 0.057) and cattle-grazed (t = 2.07, *P* = 0.12).

Bacterial communities were distinct in each experimental site (Fig. 2B). Habitat and grazing types significantly affected the bacterial community composition (Fig. 2B, Adonis: habitat type R^2^ = 0.28, *P* < 0.0001; grazing type R^2^ = 0.21, *P* < 0.0001), but there was no significant effect of midge presence (Adonis: R^2^ = 0.02, *P* = 0.496). Further, potential midge larval habitat was dominated by bacterial phyla Acidobacteria, Actinobacteria, Proteobacteria, Bacteroidetes, Chloroflexi, Firmicutes, Planctomycetes and Verrucomicrobia (Fig. 2C). Significant effects of habitat and grazing types and their interaction on abundances of Acidobacteria, Actinobacteria, Chloroflexi and Firmicutes were observed (Additional file 2: Tables S2, S3). Mean abundance of Acidobacteria was significantly lower in pond (t = −3.15, *P* = 0.006) than spring habitats while mean abundances of Chloroflexi and Firmicutes were significantly higher in pond (t =  2.19, *P* = 0.044; t = 3.30, *P* = 0.004, respectively) than spring habitats. Mean abundance of Actinobacteria was significantly higher in spring (t = 3.53, *P* = 0.002) than pond habitats. Also, abundances of Acidobacteria and Actinobacteria were significantly higher in bison-grazed sites compared to cattle-grazed (t = 7.21, *P* < 0.0001 and t = 4.62, *P* = 0.0008, respectively) and non-grazed (t = 4.28, *P* = 0.001 and t = 6.43, *P* < 0.0001, respectively) sites (Additional file 2: Table S3). Both grazing type and the interaction of habitat and grazing types significantly influenced the abundances of Planctomycetes and Proteobacteria while habitat and interactions significantly influenced Verrucomicrobia abundances. Mean abundances of Planctomycetes and Proteobacteria were significantly higher in non-grazed site compared to bison-grazed (t = 17.54, *P* < 0.0001 and t = 5.91, *P* = 0.0001, respectively) and cattle-grazed (t = 17.50, *P* < 0.0001 and t = 5.59, *P* = 0.0001, respectively) sites. Abundance of Bacteroidetes was affected by habitat and grazing types (Additional file 2: Table S2). Mean abundance was significantly higher in pond (t = 2.81, *P* = 0.012) compared to spring habitats while bison-grazed sites comprised significantly lower mean abundance than cattle-grazed (t = − 4.29, *P* = 0.001) and non-grazed (t = − 4.03, *P* = 0.002) sites (Additional file 2: Table S3).

A high percentage (42.93% of total taxa) of bacterial communities at the lowest taxonomic level were detected in > 80% of samples (irrespective of habitat and grazing types), presumably the core bacterial communities, which accounted for an average of > 95.27% of total sequence reads of each sample. Some of the highly abundant and prevalent taxa (at their lowest taxonomic level, i.e., genus) included were unclassified Bacteroidetes, unclassified Chloroflexi, unclassified Acidobacteria Gp16, unclassified Spartobacteria, unclassified α-, β-, γ-, δ- Proteobacteria, *Luteolibacter* and *Gailella* (Additional file 2: Table S4). The abundances of these taxa were also significantly influenced by habitat, grazing types and their interaction (Additional file 2: Table S4). Pond comprised significantly higher abundances of unclassified Bacteroidetes (t = 5.02, *P* = 0.0001), Actinobacteria_Gp16 (t = 5.45, *P* = 0.0001), unclassified Chloroflexi (t = 2.18, *P* = 0.044), *Luteolibacter* (t = 4.58, *P* = 0.0003) than spring habitats. Abundances were significantly higher in the springs for *Gaiella* (t = 6.18, *P* < 0.0001), unclassified Spartobacteria (t = 3.39, *P* = 0.003), unclassified α-Proteobacteria (t = 4.83, *P* = 0.0002), β-Proteobacteria (t = 4.08, *P* = 0.0009), γ-Proteobacteria (t = 8.12, *P* < 0.0001), δ-Proteobacteria (t = 2.27, *P* = 0.037) than the pond habitats. Also, non-grazed sites consisted of significantly higher abundances of unclassified α-, β-, γ-, δ- Proteobacteria than bison-grazed (t = 8.67, 4.41, 5.84, 7.09; *P* < 0.0001, = 0.0012, = 0.0001, < 0.0001, respectively) and cattle-grazed (t = 13.09, 6.12, 8.41, 5.32; *P* < 0.0001, < 0.0001, < 0.0001, = 0.0002, respectively) sites.

### Protistan diversity and communities

**Fig. 3:**
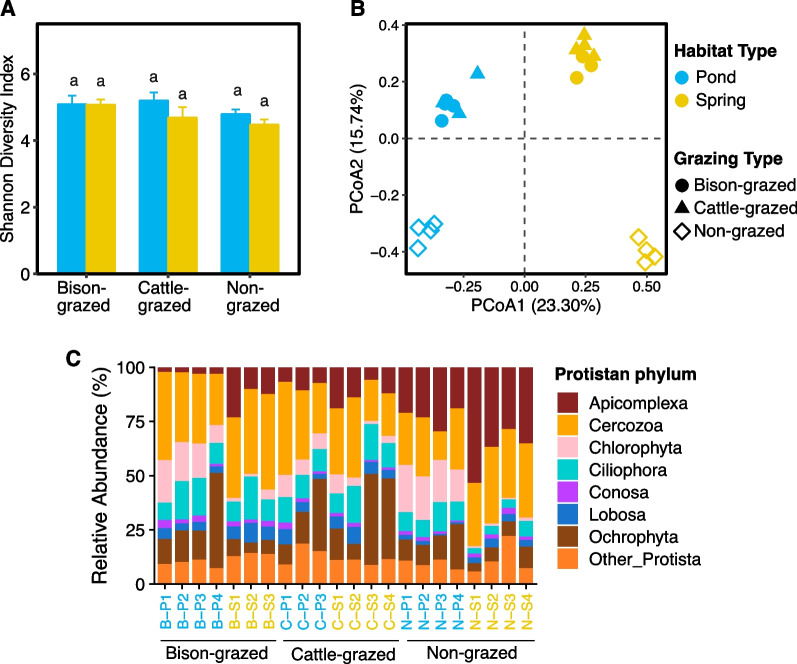
Protistan diversity and community composition of potential midge habitat soil. **A** Shannon diversity index in disturbed (bison- and cattle-grazed) and undisturbed (non-grazed) pond and spring habitats. The error bars are standard errors of means. The different letters indicate significant differences between sampling sites (*P* ≤ 0.05). **B** Protistan community composition in each sample. The first and second axes of Principal Co-ordinates Analysis illustrates Euclidean distances between samples. **C** Protistan community composition (phyla). Relative abundance for each sample is shown, and sample names are color coded based on habitat types on the horizontal axis (pond = blue and spring = yellow)

The 18S rRNA gene sequencing assigned to microbial eukaryotes comprised 1,909,975 sequence reads. Of the total eukaryote sequences, only 753,722 sequence reads were assigned to protists which clustered into 2,066 OTUs. The number of OTUs per sample ranged from 443 to 828. Approximately 59.1% of total OTUs were shared between habitats and 40.6% among grazing types. Protistan species richness was significantly affected by grazing type (F_(2,16)_ = 5.15, *P* = 0.019) but not by habitat type or interaction of habitat and grazing types. Mean species richness was significantly higher in bison-grazed than non-grazed (t = 3.01, *P* = 0.021) sites. Also, there was no significant effect of habitat, grazing types or their interaction on protistan Shannon diversity index (Fig. 3A) and Pielou’s evenness. Consequently, no differences were observed in mean Shannon diversity or Pielou’s evenness between any sampling site (Additional file 2: Table S1). Protistan beta diversity was significantly affected by grazing type (F_(2,16)_ = 7.95, *P* = 0.0040) and the interaction of grazing and habitat (F_(2,16)_ = 5.64, *P* = 0.0140) types. Mean beta diversity was significantly higher in non-grazed compared to bison-grazed (t = 3.53, *P* = 0.007) and cattle-grazed (t = 2.81, *P* = 0.031) sites (Additional file 2: Table S1).

Protistan community composition was distinct in each site and more similar if they were from similar habitat and/or grazing types (Fig. 3B). Effects of both habitat (Adonis: R^2^ = 0.26, *P* < 0.0001) and grazing (Adonis: R^2^ = 0.21, *P* < 0.0001) types were significant on protistan community composition, but there was no significant effect of midge presence. Also, the dominant protistan phyla in midge larval habitats were Cercozoa, Apicomplexa, Ochrophyta, Ciliophora, Chlorophyta and Lobosa (Fig. 3C). Habitat and grazing types significantly affected the abundances of Apicomplexa (*P* = 0.0006 and < 0.0001, respectively) and Lobosa (*P* = 0.0034 and 0.0009, respectively) but not their interaction (Additional file 2: Tables S2, S3). Abundances of Apicomplexa (t = 4.09, *P* = 0.0008) and Lobosa (t = 3.45, *P* = 0.003) were significantly higher in springs compared to pond habitats while abundance of Apicomplexa was significantly higher in non-grazed than cattle-grazed (t = 6.42, *P* < 0.0001) and bison-grazed (t = 6.91, *P* < 0.0001) sites. Only grazing type significantly influenced the abundance of Ciliophora (*P* = 0.012), which was significantly higher in bison-grazed (t = 3.07, *P* = 0.019) and cattle-grazed (t = 2.65, *P* = 0.043) than non-grazed sites. Abundances of Chlorophyta were significantly affected by habitat type (*P* < 0.0001) and interaction of habitat and grazing types (*P* = 0.001). Abundance of Chlorophyta was significantly higher in pond than spring habitats (t = 8.96, *P* < 0.0001). We observed no significant effects of habitat, grazing types or their interactions on abundances of remaining protistan phyla (Additional file 2: Tables S2, S3).

Like bacterial communities, “core” protistan communities also were identified, although a lower percentage (23.42%) of total taxa (at their lowest taxonomic level) contributed to this “core community”. In average 69.34% of total protistan sequences were represented by those core communities which included unclassified Cercozoa, unclassified Sandonidae, unclassified Rhogostoma, *Paracercomonas*, unclassified Peronosporales and *Sandona* (Additional file 2: Table S5).

Trophic status was assigned for each taxon within the protistan communities using their lowest taxonomic level which included consumers, consumers/plant pathogens, parasites, photoautotrophs, photoautotrophs/consumers, symbionts and unassigned. The majority of assigned trophic categories were consumers (45.74% of total abundance, Additional file 1: Fig. S1A), parasites (22.90% Additional file 1: Fig. S1B) and photoautotrophs (24.04%, Additional file 1: Fig. S1C). Other trophic categories consumers/plant pathogens, photoautotrophs/consumers, symbionts and unassigned comprised low abundances (0.66%, 1.95%, 0.23% and 4.50%, respectively) of the total protistan community (Additional file 1: Fig. S1D–G). Further analysis was performed on highly abundant (> 10% of total abundance) trophic groups only (consumers, parasites and photoautotrophs). Grazing type (*P* = 0.0018) significantly affected consumers abundance while habitat type (*P* = 0.0005) and the interaction of habitat and grazing (*P* = 0.012) types significantly influenced photoautotrophs abundance. Consumers abundance was significantly higher in both bison-grazed (t = 4.51, *P* = 0.001) and cattle-grazed (t = 2.61, *P* = 0.047) than non-grazed sites. Photoautotrophs abundance was significantly higher in pond (t = 4.19, *P* = 0.0007) compared to spring habitats. Both habitat (*P* = 0.0002) and grazing (*P* < 0.0001) types significantly affected parasites abundance. Interestingly, parasites abundance was significantly lower in pond (t = − 4.46, *P* = 0.0004) than spring habitats. Additionally, non-grazed sites contained significantly higher parasites abundance compared to bison-grazed (t = 7.04, *P* < 0.0001) and cattle-grazed (t = 6.56, *P* < 0.0001) sites. Moreover, greater abundance of the protistan phyla Cercozoa, Cilliophora, Lobosa and Conosa were observed in the consumers group (Additional file 1: Fig. S2A). The phyla Chlorophyta and Ochrophyta dominated the photoautotrophs groups, while the Apicomplexa and Pseudofungi dominated the parasites groups (Additional file 1: Fig. S2B, C).

Habitat type, grazing type and their interaction had no significant effects on consumer’s Shannon diversity index (Additional file 1: Fig. S3A) while habitat type significantly influenced the Shannon diversity index of photoautotrophs (F_(1,16)_ = 4.70, *P* = 0.046, Additional file 1: Fig. S3C). Mean Shannon diversity index of photoautotrophs was significantly higher in pond (t = 2.16, *P* = 0.04) than spring habitats. Grazing (F_(2,16)_ = 9.11, *P* = 0.002) and habitat (F_(1,16)_ = 11.71, *P* = 0.003) types affected the Shannon diversity index of parasites (Additional file 1: Fig. S3B). Mean Shannon diversity index of parasites was significantly lower in non-grazed compared to bison-grazed (t = − 3.63, *P* = 0.006) and cattle-grazed (t = − 3.70, *P* = 0.005) sites. Also, parasites Shannon diversity index was significantly higher in pond (t = 3.51, *P* = 0.003) than spring habitats. Unlike alpha diversity, habitat and grazing types significantly influence the community composition of consumers (Adonis: habitat, R^2^ = 0.13, *P* = 0.001; grazing, R^2^ = 0.26, *P* = 0.001), photoautotrophs (Adonis: habitat, R^2^ = 0.25, *P* = 0.001; grazing, R^2^ = 0.18, *P* = 0.001), and parasites (Adonis: habitat, R^2^ = 0.28, *P* = 0.001; grazing, R^2^ = 0.25, *P* = 0.001) but not the presence of midges (*P* > 0.05, each) (Additional file 1: Fig. S3D–F).

### Fungal diversity and communities

**Fig. 4:**
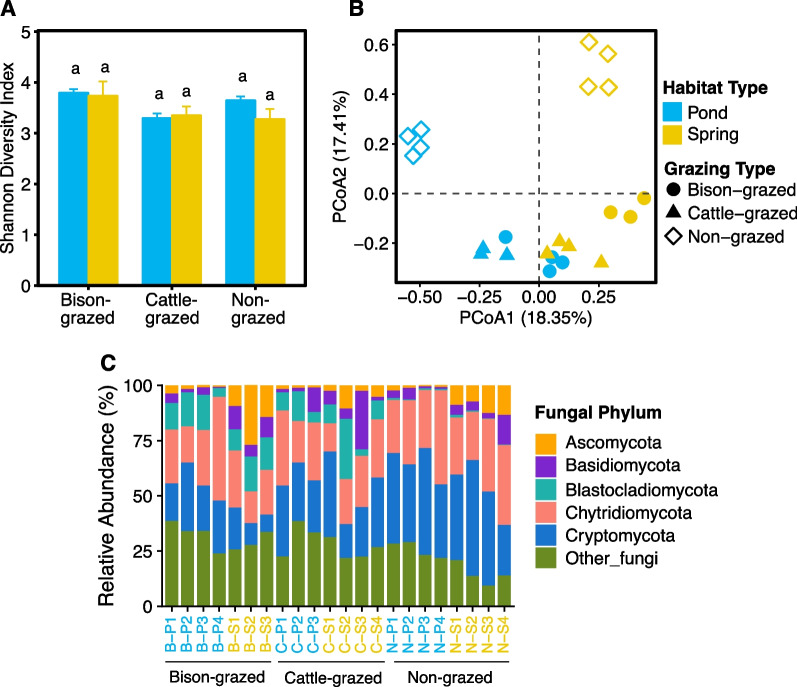
Fungal diversity and community composition of potential midge habitat soil. **A** Shannon diversity index in disturbed (bison- and cattle-grazed) and undisturbed (non-grazed) pond and spring habitats. The error bars are standard errors of means. The different letters indicate significant differences between sampling sites (*P* ≤ 0.05). **B** Fungal community composition in each sample. The first and second axes of Principal Co-ordinates Analysis shows Euclidean distances between samples. **C** Fungal community composition (phyla). Relative abundance for each sample is shown, and sample names are color coded based on habitat types on the horizontal axis (pond = blue and spring = yellow)

Out of 1,909,975 18S rRNA sequences, only 146,481 sequence reads were assigned to true fungi. Those sequence reads were clustered into 435 OTUs. The number of OTUs per sample ranged from 74 to 149. Fungal species richness was significantly affected by grazing (F_(2,16)_ = 7.27, *P* = 0.0057) but not by habitat (F_(1,16)_ = 0.20, *P* = 0.6634) types or their interaction (F_(2,16)_ = 0.70, *P* = 0.5109). Mean species richness was significantly higher in bison-grazed than non-grazed (t = 3.63, *P* = 006) sites. Similarly fungal Shannon diversity index was significantly influenced by grazing type (F_(2,16)_ = 4.05, *P* = 0.038, Fig. 4A). Shannon diversity index was significantly higher in bison-grazed sites than cattle-grazed (t = 2.73, *P* = 0.036) sites. There was no significant effect of habitat and grazing types and their interaction on fungal Pielou’s evenness. Only habitat significantly influenced the fungal beta diversity (F_(1,16)_ = 10.35, *P* = 0.0054), which was significantly higher in spring (t = 3.13, *P* = 0.006) than pond habitats.

The composition of fungal communities across soil samples were more similar if they were from similar habitat and grazing types (Fig. 4B). Both habitat (Adonis: R^2^ = 0.25, *P* < 0.0001) and grazing (Adonis: R^2^ = 0.19, *P* < 0.0001) types significantly affected the fungal community composition but not midge presence (Adonis: R^2^ = 0.02, *P* = 0.58). Interestingly, fungal communities were dominated by Chytridiomycota (26.07%) and Cryptomycota (28.79%). Comparatively low abundances were observed for Ascomycota (5.90%), Basidomycota (5.61%) and Blastocladiomycota (7.54%) (Additional file 2: Table S3). Grazing significantly affected the abundances of Cryptomycota (Additional file 2: Table S2) and Blastocladiomycota (Additional file 2: Table S2). Abundance of Cryptomycota was significantly lower in bison-grazed (t = − 4.97, *P* = 0.0004) and cattle-grazed (t = − 2.78, *P* = 0.033) compared to non-grazed sites. Interestingly, abundance of Blastocladiomycota was significantly higher in both bison-grazed (t = 4.14, *P* = 0.002) and cattle-grazed (t = 3.35, *P* = 0.010) sites than non-grazed sites. Abundance of Ascomycota was influenced by both habitat (Additional file 2: Table S2) and grazing (Additional file 2: Table S2) types, where abundance was significantly lower in pond than spring (t = − 5.67, *P* < 0.0001) habitats. No significant effects of habitat and/or grazing were observed for Basidiomycota and Chytridiomycota abundances (Additional file 2: Tables S2, S3).

### Metazoan diversity and communities

**Fig. 5:**
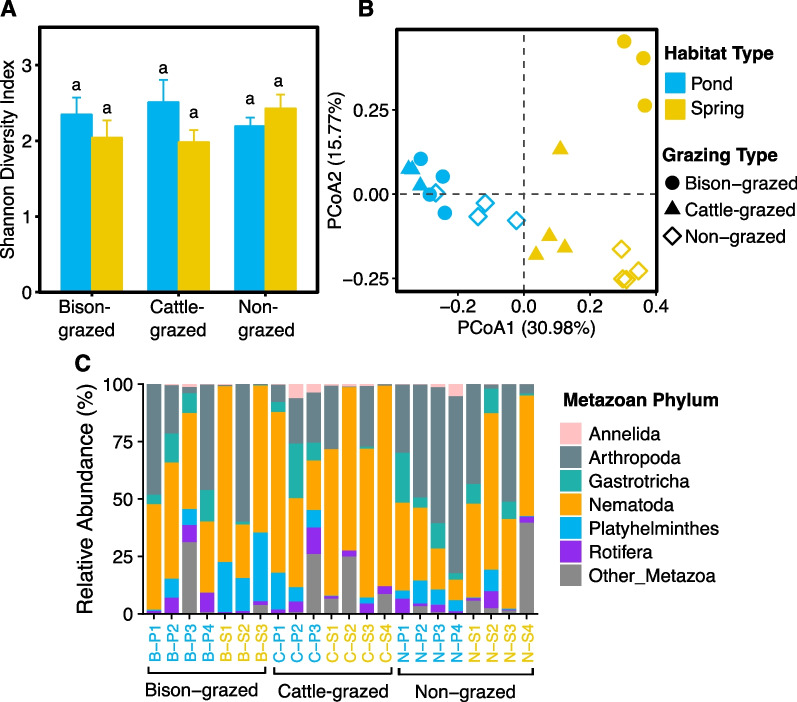
Metazoan diversity and community composition of potential midge habitat soil. **A** Shannon diversity index in disturbed (bison- and cattle-grazed) and undisturbed (non-grazed) pond and spring habitats. The error bars are standard errors of means. The different letters indicate the significant differences between sampling sites (*P* ≤ 0.05). **B** Metazoan community composition in each sample. The first and second axes of Principal Co-ordinates Analysis depicts Bray–Curtis distances between samples. **C** Metazoan community composition (phyla). Relative abundance for each sample is shown, and sample names are color coded based on habitat types on the horizontal axis (pond = blue and spring = yellow)

Approximately 23.72% (453,039) of 18S rRNA amplicon sequence reads were classified as Metazoa. There were 218 OTUs distributed in 22 samples ranging from 21 to 65 per sample. Metazoan species richness was significantly affected by grazing type (F_(2,16)_ = 18.95, *P* < 0.0001) and interaction of habitat and grazing types (F_(2,16)_ = 4.37, *P* = 0.0307) but not by habitat type. Mean species richness was significantly higher in non-grazed compared to bison-grazed (t = 5.54, *P* = 0.0001) and cattle-grazed (t = 4.80, *P* = 0.0005) sites. However, no significant difference between mean species richness was observed between bison-grazed and cattle-grazed sites. Habitat, grazing types and their interaction had no significant influence on the metazoan Shannon diversity index (Fig. 5A) and Pielou’s evenness. No difference was observed between mean Shannon diversity index (Fig. 5A) or metazoan Pielou’s evenness between sampling sites. Interestingly, habitat type (F_(1,16)_ = 15.99, *P* = 0.0010) and the interaction of grazing and habitat types (F_(2,16)_ = 11.75, *P* = 0.0007) had a significant influence on metazoan beta diversity. Metazoan beta diversity was significantly lower in pond (t = − 3.95, *P* = 0.001) than spring habitats.

The composition of potential midge larval habitat soil metazoan communities was more similar if samples were from the same or similar types of natural habitat (Fig. 5B). Habitat (Adonis: R^2^ = 0.29, *P* < 0.0001) and grazing (Adonis: R^2^ = 0.17, *P* = 0.0002) types significantly affected the metazoan community composition but not by midge presence (Adonis: R^2^ = 0.01, *P* = 0.89). Metazoan communities in soils from potential midge larval habitats were Nematoda, Arthropoda, Rotifera, Platyhelminthes, Gastrotricha and Annelida (Fig. 5C). Habitat type significantly affected the abundances of Annelida, Gastrotricha and Nematoda (Additional file 2: Table S2). Compared to spring habitats, pond habitats had significantly higher abundances of Annelida (t = 2.48, *P* = 0.24) and Gastrotricha (t = 3.17, *P* = 0.006), but significantly lower abundances of Nematoda (t = − 3.11, *P* = 0.007). Abundance of Platyhelminthes was significantly influenced by grazing type and interaction of habitat and grazing types (Additional file 2: Tables S2, S3), being higher in bison-grazed sites than cattle-grazed (t = 3.16, *P* = 0.015) and non-grazed (t = 3.66, *P* = 0.005) sites. Abundance of Rotifera and Arthropoda were not affected by grazing type, habitat or their interaction.

### Relationships between microbial diversity, communities, soil properties and midge presence

Significant negative correlations between midge presence and soil properties (TN (r = − 0.61), TC (r = − 0.49) and OM (r = − 0.52)) were observed. Moreover, TN, TC and OM were significantly greater in soil lacking *Culicoides* spp. than soil with *Culicoides* spp. (Additional file 1: Fig. S4). Although bacterial diversities (species richness, Shannon diversity index, Pielou’s evenness and beta diversity) were negatively correlated with the presence of *Culicoides,* only the correlation to beta diversity was statistically significant (Table 1). There were no significant correlations between soil properties (TN, TC and OM) and bacterial diversity. Protistan species richness was positively correlated with *Culicoides* presence while beta diversity was negatively correlated (Table 1). Further, beta diversity of protistan trophic groups (consumers and parasites) was negatively correlated with presence of *Culicoides*. None of the soil properties were significantly correlated with protistan diversities. Significant negative correlations were observed between soil properties (TN and OM) and consumers species richness. TN, TC and OM was also negatively correlated with Shannon diversity index and Pielou’s evenness of parasites. A positive correlation was observed between fungal species richness and *Culicoides* presence. Soil properties (TN, TC, OM) negatively correlated with fungal species richness (Table 1). *Culicoides* presence was negatively correlated with metazoa species richness; however, soil properties (TN and OM) were positively correlated with metazoa species richness. Soil TC was negatively correlated with metazoan Pielou’s evenness (Table 1).

**Table 1:** Correlations between microbial (bacterial, fungal, metazoan and protistan) alpha- and beta-diversities, soil properties and presence of biting midges

Kingdom		Diversity	*Culicoides*	TN	TC	OM
Bacteria		Richness	− 0.19	0.39	0.39	0.38
		Shannon	− 0.26	0.28	0.37	0.3
		Evenness	− 0.22	0.13	0.24	0.16
		Beta diversity	− **0.43**	0.23	0.04	0.22
Protista						
	Overall	Richness	**0.49**	− 0.33	− 0.35	− 0.38
		Shannon	0.17	− 0.26	− 0.37	− 0.23
		Evenness	0	− 0.18	− 0.3	− 0.12
		Beta diversity	− **0.63**	0.33	0.23	0.4
	Photoautotroph	Richness	0.21	− 0.1	− 0.19	− 0.22
		Shannon	− 0.03	0.12	− 0.11	0.17
		Evenness	− 0.18	0.2	− 0.04	0.31
		Beta diversity	− 0.24	0.01	0.01	0.17
	Consumer	Richness	**0.52**	− **0.43**	− 0.39	− **0.43**
		Shannon	**0.38**	− 0.31	− 0.28	− 0.23
		Evenness	0.15	− 0.13	− 0.13	− 0.04
		Beta diversity	− **0.67**	0.37	0.26	0.39
	Parasite	Richness	0.05	0.33	0.29	0.31
		Shannon	**0.42**	− **0.62**	− **0.65**	− **0.59**
		Evenness	0.39	− **0.67**	− **0.68**	− **0.64**
		Beta diversity	− **0.48**	0.12	0.06	0.21
Fungi		Richness	**0.49**	− **0.44**	− **0.46**	− **0.44**
		Shannon	0.14	0.03	− 0.13	− 0.03
		Evenness	− 0.05	0.22	0.04	0.16
		Beta diversity	− 0.36	0.07	0.1	0.13
Metazoa		Richness	− **0.5**	**0.51**	0.34	**0.54**
		Shannon	− 0.15	− 0.11	− 0.26	− 0.1
		Evenness	0.08	− 0.34	− **0.43**	− 0.35
		Beta diversity	0.03	− 0.18	− 0.24	− 0.06

Significant Pearson correlation coefficients are shown in bold

*Culicoides* = presence of *Culicoides* spp., *TN* total nitrogen, *TC* total carbon, *OM* organic matter

Abundances of bacterial phyla (Proteobacteria and Planctomycetes) were negatively correlated with *Culicoides* presence while Firmicutes correlated positively (Table 2). Soil properties (TN, TC and OM) negatively affected the abundances of Actinobacteria and Firmicutes but positively impacted abundance of Planctomycetes and Proteobacteria (Table 2). Abundance of protistan phylum Apicomplexa was negatively correlated with *Culicoides* presence. Soil properties (TN, TC and OM) positively affected the abundance of Apicomplexa, but TC was negatively associated with the abundances of phyla Cercozoa and Conosa. Abundance of the fungal phylum Blastocladiomycota was positively correlated with *Culicoides* presence (Table 2). Soil properties (TN, TC and OM) negatively affected the abundance of Blastocladiomycota but positively impacted the abundance of Cryptomycota. Only CN was positively correlated with the abundance of Basidiomycota (Table 2).

**Table 2:** Correlations between relative abundance of microbial phyla from different kingdom, soil properties and presence of biting midges

Kingdom	Phylum	*Culicoides*	TN	TC	OM
Bacteria	Acidobacteria	− 0.08	− 0.22	− 0.29	− 0.16
	Actinobacteria	0.36	− **0.62**	− **0.67**	− **0.51**
	Bacteroidetes	− 0.05	0.26	0.34	0.18
	Chloroflexi	0.21	0.1	0.3	− 0.04
	Firmicutes	**0.47**	− **0.5**	− **0.46**	− **0.46**
	Planctomycetes	− **0.69**	**0.93**	**0.78**	**0.92**
	Proteobacteria	− **0.6**	**0.8**	**0.86**	**0.7**
	Verrucomicrobia	− 0.06	0.11	− 0.04	0.1
Protista	Apicomplexa	− **0.47**	**0.69**	**0.59**	**0.73**
	Cercozoa	0.06	− 0.28	− **0.42**	− 0.13
	Chlorophyta	− 0.04	0.24	0.14	0.12
	Ciliophora	0.4	− 0.36	− 0.2	− 0.38
	Conosa	0.06	− 0.31	− **0.46**	− 0.23
	Lobosa	0.4	− 0.47	− 0.34	− 0.36
	Ochrophyta	0.3	− 0.4	− 0.21	− 0.5
Fungi	Ascomycota	− 0.15	0.08	0.06	0.22
	Basidiomycota	0.08	− 0.13	0.03	− 0.14
	Blastocladiomycota	**0.47**	− **0.56**	− **0.47**	− **0.51**
	Chytridiomycota	− 0.25	0.18	0.04	0.13
	Cryptomycota	− 0.39	**0.52**	**0.5**	**0.44**
Metazoa	Annelida	0.01	− 0.04	− 0.08	− 0.07
	Arthropoda	− 0.14	0.4	0.35	0.37
	Gastrotricha	− 0.13	0.06	− 0.12	0.06
	Nematoda	0.21	− 0.35	− 0.18	− 0.35
	Platyhelminthes	− 0.09	0	− 0.14	0.06
	Rotifera	0.09	− 0.33	− 0.38	− 0.37

Significant Pearson correlation coefficients are shown in bold

*Culicoides* = presence of *Culicoides* spp., TN = total nitrogen, TC = total carbon, OM = organic matter

Abundance of some bacterial taxa (classified at the lowest taxonomic level) such as unclassified Betaproteobacteria, Deltaproteobacteria, Gammaproteobacteria, Acidobacteria Gp17, unclassified Rhodobacteriaceae, *Hyphomicrobium* were negatively correlated with *Culicoides* presence but unclassified Anaerolineaceae, Chloroflexi, *Clostridium*, *Sphingomonas* and unclassified Planococcaceae were positively correlated (Table 3). Interestingly, soil properties (TN, TC and OM) positively affected the abundances of unclassified Betaprotobacteria, Bacteroidetes, Deltaproteobacteria, Gammaproteobacteria, Acidobacteria Gp17, unclassified Rhodobacteriaceae and *Hyphomicrobium* and negatively affected the abundances of unclassified Bacillaceae, Intrasporangiaceae, Planococcaceae and *Sphingomonas* (Table 3). Abundances of protistan taxa such as *Prismatosphora* and unclassified Oomycota were negatively correlated with *Culicoides* presence while unclassified Nolandellidae and NC12B lineage positively correlated (Table 3). Soil properties (TN, TC and OM) negatively affected the abundances of unclassified Chlamydomonadales, Glissomonadida, NC12B lineage and *Prorodon* while abundances of *Syncystis* and *Prismatospora* were positively affected (Table 3).

**Table 3:** Correlations between relative abundance of most prevalent (> 80%) microbial (bacteria and protista) taxa at lowest taxonomic levels, soil properties and presence of biting midges

Kingdom	Lowest taxonomic level	*Culicoides*	TN	TC	OM
Bacteria	Acidobacteria Gp6	− 0.11	− 0.17	− 0.19	− 0.07
	Rhizobiales	− 0.25	0.02	− 0.23	0.13
	Anaerolineaceae	**0.5**	− **0.4**	− 0.17	− **0.46**
	Betaproteobacteria	− **0.67**	**0.74**	**0.74**	**0.71**
	Acidobacteria Gp16	0.23	− 0.39	− **0.5**	− 0.41
	*Gaiella*	− 0.05	− 0.2	− 0.27	− 0.11
	Actinobacteria	− 0.2	− 0.12	− 0.25	− 0.05
	*Thermoleophilia*	0.05	− 0.09	− 0.2	0
	Bacillaceae	0.39	− **0.53**	− **0.59**	− **0.43**
	Chloroflexi	**0.46**	− 0.29	− 0.15	− 0.35
	*Luteolibacter*	0.02	0.02	− 0.09	0
	Steroidobacteraceae	− 0.19	**0.65**	**0.71**	**0.52**
	Bacteroidetes	0.01	**0.54**	**0.62**	**0.45**
	Subdivision3	− 0.07	0.22	0.14	0.1
	Intrasporangiaceae	**0.64**	− **0.83**	− **0.7**	− **0.85**
	Acidimicrobiales	0.06	− 0.18	− 0.31	− 0.1
	*Clostridium*_sensu_stricto	**0.41**	− 0.33	− 0.35	− 0.34
	Caldilineaceae	− 0.37	**0.81**	**0.84**	**0.67**
	Comamonadaceae	− 0.14	0.2	0.3	0.11
	Acidobacteria Gp17	− **0.78**	**0.72**	**0.67**	**0.7**
	Rhodobacteraceae	− **0.45**	**0.68**	**0.58**	**0.57**
	*Sphingomonas*	**0.52**	− **0.71**	− **0.61**	− **0.71**
	Solirubrobacterales	0.31	− **0.52**	− **0.64**	− **0.44**
	Pirellulales	− **0.54**	**0.81**	**0.64**	**0.85**
	Deltaproteobacteria	− **0.68**	**0.73**	**0.61**	**0.76**
	*Coriobacteriia*	**0.44**	− **0.45**	− **0.51**	− **0.42**
	Burkholderiales	− 0.21	**0.63**	**0.79**	**0.5**
	Gammaproteobacteria	− **0.54**	**0.5**	0.4	**0.59**
	*Hyphomicrobium*	− **0.48**	**0.62**	**0.44**	**0.58**
	Planococcaceae	**0.43**	− **0.48**	− **0.49**	− **0.43**
	Chitinophagaceae	− 0.34	**0.6**	**0.49**	**0.52**
Protista	Sandonidae	− 0.08	0.01	− 0.11	− 0.03
	*Monocystis*	− 0.24	0.4	**0.58**	0.32
	Cercozoa	0.07	− 0.14	− 0.22	− 0.09
	*Navicula*	0.27	− 0.4	− **0.46**	− **0.43**
	*Pinnularia*	0.08	− 0.1	0.19	− 0.19
	*Leidyana*	− 0.22	0.27	0.15	0.37
	*Rhogostoma*	0.19	− 0.23	− 0.11	− 0.18
	Sphaeropleales	− 0.13	0.35	0.25	0.22
	Peronosporales	0.27	− 0.36	− 0.22	− 0.27
	*Hypotrichia*	0.34	− 0.24	0	− 0.27
	*Vaucheria*	0.12	− 0.1	0.1	− 0.15
	Actinocephalidae	− 0.39	0.3	0.22	0.38
	Paracercomonas	0.06	0.05	0	0.15
	Chlamydomonadales	0.34	− **0.51**	− **0.6**	− **0.51**
	*Prismatospora*	− **0.43**	**0.81**	**0.75**	**0.69**
	Nolandellidae	**0.43**	− 0.29	− 0.2	− 0.17
	*Colpodida*	0.14	− 0.13	0.22	− 0.18
	*Pythium*	0.19	− 0.25	− 0.16	− 0.24
	*Syncystis*	− 0.29	**0.6**	**0.56**	**0.49**
	Sandonidae1	0.23	− 0.31	− **0.44**	− 0.23
	Oomycota	− **0.42**	0.35	0.31	0.33
	Anurofeca	− 0.15	0.35	0.24	0.24
	Glissomonadida	0.36	− **0.53**	− **0.57**	− **0.52**
	*Sandona*	0.25	− 0.14	− 0.19	0.04
	*Prorodon*	0.3	− **0.45**	− **0.5**	− **0.45**
	Eustigmatophyceae	0.11	− 0.09	− 0.25	− 0.14
	*Surirella*	0.3	− 0.42	− **0.42**	− **0.43**
	NC12B lineage	**0.56**	− **0.57**	− **0.61**	− **0.57**
	*Sessilida*	0.19	0.01	0.03	0.13
	*Eocercomonas*	− 0.16	0.19	0.12	0.34
	*Cercomonas*	− 0.22	0.14	− 0.01	0.26

Significant Pearson correlation coefficients are shown in bold

*Culicoides* = presence of *Culicoides* spp., TN = total nitrogen, TC = total carbon, OM = organic matter

## Discussion

This study explored the complex relationships between habitat use (disturbed (bison- and cattle-grazed) and undisturbed (non-grazed)), microbial (bacteria, protista, fungi) communities, soil small animals (metazoa), soil properties and presence of midges from natural pond and spring habitats in the Konza Prairie. One key finding was that midge presence was associated with a decrease in microbial (bacterial, protistan and fungal) beta-diversities. This phenomenon may have resulted from midge larvae feeding and thereby depleting the frequency (or abundance) of certain microbes in the soil substrate, such as taxa within the phyla Proteobacteria (Bacteria), Planctomycetes (Bacteria), and Apicomplexa (Protista) (see details in Results). Previous studies have demonstrated that dipteran larvae obligately feed upon microbes as they develop, including *Culicoides stellifer* [15], *Musca domestica* L. [12], and *Stomoxys calcitrans* L. [13]. Alternatively, midges may compete with these microbes for other resources in the substrate or may produce inhibitory substances that hinder their colonization, by unknown mechanisms that warrant further exploration. Interestingly, some microbial communities such as abundances of phyla Firmicutes (Bacteria), Blastocladiomycota (Fungi), and associated lower taxa (see Results) increased when midge larvae were present, indicating that grazing (depletion) or inhibition of some microbial taxa promotes abundance of others as we have previously observed in house fly larval grazing on manure [14].

Soil properties differed between grazed and non-grazed prairie sites. Lower TN, TC and OM in bison and cattle grazed sites compared to non-grazed sites may be due to the presence of large mammals, whose grazing depletes the above ground biomass. In turn, this results in reduced photosynthesis and carbon sequestration which consequently lowers the level of soil TN, TC and OM in the landscape. Prior studies have demonstrated higher carbon in soils from non-grazed virgin grasslands than livestock-grazed grasslands in the Northern Great Plains [32], and greater soil, carbon, and nitrogen content in soils from non-grazed sites compared to moderate and intense sheep-grazed sites [33]. Higher TC was observed in soil from springs than pond habitats but there was no difference in TN or OM. Those variations in soil properties of different habitat types may be associated with diverse biotic and abiotic factors of the distinct and uniquely situated experimental sites.

Greater midge prevalence in both bison and cattle-grazed pond and spring habitats than in non-grazed habitats can be attributed to adult midge requirements, such as proximity to ruminant hosts that serve as blood meals for anautogenous females. Livestock farm habitats harbored a greater number of *C. obsoletus* and *C. scoticus* than wetland and peri-urban habitats in Europe [34]. Similarly, a white-tailed deer farm (game preserve) harbored a greater number of *C. stellifer* than alternate sites in Florida [35]. Total carbon, TN and OM was significantly reduced in the presence of *Culicoides* spp. which is in contradiction with a previous study that showed a positive correlation of number of total midges (including different *Culicoides* species) with soil organic matter [36]. Another study that evaluated the role of habitat in emergence of *Culicoides* spp. from commercial farms showed no clear association of soil properties on midge abundance and/or emergence [9]. These variable findings indicate that in some habitats (e.g., in the Konza Prairie) midge larvae feed upon and deplete decaying organic matter and nutrients, while in other habitats they do not.

### Effects of habitat and disturbance on microbial community composition and diversity

Potential midge larval habitat soil bacterial communities were dominated by phyla Proteobacteria, Actinobacteria, Acidobacteria, Firmicutes, Chloroflexi, Bacteroidetes and Verrucomicrobia, which have been previously reported from conventional cropland, restored grasslands and annually burned native tallgrass prairie at the Konza Prairie Biological Station (KPBS), Kansas [37], where our samplings sites are also situated. The abundance of most of those bacterial phyla were affected differently by habitat type and disturbances (grazing). That variability might be explained by variability in additional soil physico-chemical properties (which we did not measure), as well as other biotic and abiotic factors that were not captured in our study. Several taxa within these phyla (see Results) also were influenced by habitat and grazing types, but because they were found in > 80% samples these taxa potentially comprise a “core microbiome” across our samples. A previous study showed high prevalence of taxa affiliated to phylum Verrucomicrobia reported from similar tallgrass prairie habitats [38] and KPBS [37, 39] which is in concordance with our study results. The core microbiome represented > 95% of total abundance (sequence reads) in the samples and was likely due to the similar nature of the soil samples, which were all selected from banks of ponds and streams, i.e., presumptive semi-aquatic midge breeding sites [40–42].

Soil bacterial community composition was associated with habitat and grazing type, which is in accordance with a previous study at KPBS that showed the land management (e.g., grazing type) strongly correlated with soil microbial community composition [37]. Interestingly, midge presence did not influence bacterial community composition which is likely due to sampling time, which was during cooler months, September to December, when midge activity decreases and breeding behavior diminishes [43]. Alpha diversity indices were variably impacted by habitat and grazing type, where there was no impact on species richness but significant impacts on Shannon diversity index and Pielou’s evenness (just by grazing type). These results suggest that the number of bacterial species were somewhat similar across samples, but the abundances were highly variable between samples within a habitat or grazing type. Such variability could be explained by various environmental stressors including soil properties such as pH [44], moisture and precipitation [45] that can profoundly influence bacterial communities.

As observed with soil bacterial community composition, protistan communities within individual samples were strongly associated with habitat and land management (grazing) type but not with midge presence in the soil, with the latter lack of effect being likely attributable to diminished midge abundance and activity (discussed above). Protistan communities, specifically the phyla Apicomplexa and Lobosa, were significantly impacted by both habitat and grazing types, with a higher relative abundance of Apicomplexa in non-grazed and spring habitats than in grazed ponds. Several taxa of Apicomplexa are known for their parasitic behavior to diverse organisms, such as *Leidyana* sp. parasites of insects [46] and *Monocystis* sp. parasites of Earthworms [47]. The relative abundances of these taxa had a negative association to the relative abundance of Arthropoda and positive association with Nematoda (result not shown), indicating an intriguing possibility that some correlations are related to these taxa parasitizing arthropods or other soil invertebrates. Moreover, the relative abundance of Ciliophora was higher in grazed sites than non-grazed sites, suggesting underlying factors influence the abundance of Ciliophora in those habitats. Ciliophora are key consumers in different ecosystems, preying on bacteria, other protozoa, unicellular algae, occasionally rotifers and microzooplankton [48]. Irrespective of grazing type, abundance of the ciliate *Prorodon* sp., was higher in ponds than in springs, possibly due to their ability to thrive in stagnant water/soil interfaces of ponds which have been shown as optimal habitats [49]. In contrast, other consumers such as Nolandellidae (Lobosa) were more often observed in springs rather than pond habitats, probably attributable to their preferred trophic associations.

Although both habitat type and grazing shaped the structure of soil fungal communities, variation in fungal community composition was higher in non-grazed sites than grazed sites, and fungal community composition was clearly separated between pond and spring habitats. However, fungal community compositions in soil samples of both grazing regimens (bison and cattle) were more similar to each other than those found in non-grazed sites. This result supports the idea that grazing activity by mammals shapes the fungal (and other microbial) communities and their composition in grasslands [50, 51]. Grazing significantly affects the micro and macro-nutrients in the soil which consequently alters the microorganisms and small soil invertebrates residing in the soil. Fungal diversity also varied, albeit non-significantly, across sites where species richness in non-grazed sites (both ponds and springs) was lower compared to grazed sites. Animals can likely introduce additional microbial taxa either directly from excreta (e.g., manure) or indirectly by passive carriage and dispersal as they wander and graze. Also, grazers may favor certain fungal species to flourish while others diminish via altering soil microenvironment which likely decreased soil fungal diversity and altered community composition [52].

Phyla Cryptomycota and Chytridiomycota were the most abundant and highly prevalent soil fungal communities in potential midge larval habitats, which is not surprising as our samples were from banks of ponds and springs and these phyla have previously been reported in many aquatic habitats [53, 54]. Although highly prevalent, the abundances of phyla Basidiomycota and Ascomycota were highly variable across the site. These phyla are considered basal soil fungal communities and have been previously reported from the tallgrass prairie [55]. Interestingly, the relative abundance of phylum Blastocladiomycota was lower in both non-grazed sites than grazed sites. Species of phylum Blastocladiomycota are known to be prevalent in soil and water, and include several saprotrophs, pathogens of aquatic Dipteran larvae, Nematoda and Crustacea [56, 57]. Therefore, the low abundance of those fungi in non-grazed sites could have been due to biotic (microbe and small invertebrate) and abiotic factors associated with those sites.

Nematoda, Arthropoda and Gastrotricha were highly prevalent phyla represented in the soil metazoan communities. Although habitat type affected the relative abundances of Nematoda and Gastrotricha, the relative abundances of all metazoan phyla within a habitat varied among samples, which may be attributable to sampling methods, relative body size of these multicellular organisms compared to unicellular microbes, dispersal [58] and other behaviors, and trophic status (predator, prey). For instance, several groups of soil nematodes are known predators of bacteria and fungi [59], and therefore might compete with midges for these prey items in the substrate. Conversely, some prey species of nematodes may disperse in response to predators including midges [12, 15] and other Arthropods in the habitat [60], which is supported by the negative correlations of these phyla. Another highly prevalent phylum Gastrotricha contains organisms that consume detritus, protists and bacteria [61]. Gastrotricha predation may underlie negative correlations between abundance of this phylum and abundances of several protistan and bacterial taxa.

Our study design has some limitations that need to be addressed. Samples were collected in different months (September to December) and were considered replicates; however, environmental factors (e.g., temperature, precipitation, vegetation) likely changed across the sampling period and introduced variability which was not captured in our analyses. Additionally, non-grazed sites were likely visited by other herbivores such as white-tailed deer and other small animals present at the KPBS, which may have additionally affected the microbial and eukaryal communities. Although this study utilized amplicon sequencing of highly conserved V3-V4 and V4 region of 16S (~ 450 bp) and 18S rRNA (~ 417 bp) genes to characterize bacterial and eukaryal communities respectively, the short sequence reads generated could not resolve taxonomic affiliation of some individual taxa at their lower taxonomic levels [62]. Moreover, this study used a single pair of primers for characterizing three different eukaryotic kingdoms (protista, fungi and metazoa) which could limit identification accuracy and capturing diversity [63, 64]. Thus, more fine scale sampling across an entire active season of midge species as well as utilizing both culture and culture-independent shotgun metagenome or long-amplicon sequencing methods could provide more insight into the role of habitat, disturbance and other abiotic factors on microbial communities as well as midge prevalence in future studies.

## Conclusions

This study demonstrated that habitat type and disturbance (i.e., grazing type) and soil properties (TN, TC, OM) strongly shaped soil bacterial, protistan, fungal and metazoan community compositions, diversities and midge presence. Abundances of specific microbial taxa, notably *Hyphomicrobium*, unclassified β-, δ- and γ-proteobacteria (Proteobacteria, Bacteria), Pirellulales (Planctomycetes, Bacteria), and *Prismatosphora* (Apicomplexa, Protista) decreased while the abundance of *Clostridium* sensu stricto (Firmicutes, Bacteria) and Nolandellidae (Amoebozoa, Protista) increased in the presence of midges in the habitat. Prevalence of midges mainly in disturbed (grazed by cattle or bison) sites indicates that midges prefer to breed and shelter in a habitat with greater accessibility to animals, who are a potential source of obligate blood meals for anautogenous females. These results provide the first detailed insights, to our knowledge, into the microbial communities, soil properties and prevalence of midges in suspected midge larval habitats from a protected natural prairie. Further studies are required to understand the role of specific microbes on midge larval survival and fitness in natural habitats which can be potentially used to predict and then mitigate midge habitat use by developing novel larval habitat management methods.

## Supplementary Information

**Additional file 1: Fig. S1.** Trophic groups of soil protistan communities. Mean relative abundance of (A) Consumers, (B) Parasites, (C) Photoautotrophs, (D) Photoautotrophs/Consumers, (E) Consumers/Plant Pathogens, (F) Symbionts, and (G) Unassigned groups in disturbed (bison- and cattle-grazed) and undisturbed (non-grazed) pond and spring habitats. The error bars are standard errors of the means. **Fig. S2.** Soil protistan trophic group communities. Relative abundances protistan phyla (A) Consumers, (B) Parasites, (C) Photoautotrophs in disturbed (bison- and cattle-grazed) and undisturbed (non-grazed) pond and spring habitats. **Fig. S3.** Soil protistan trophic group diversity and community composition. Shannon diversity index of (A) Consumers, (B) Parasites, (C) Photoautotrophs in disturbed (bison- and cattle-grazed) and undisturbed (non-grazed) pond and spring habitats. The error bars are standard errors of means. The different letters indicate the significant differences between sampling sites (*P* ≤ 0.05). Protistan trophic groups community composition (D) Consumers, (E) Parasites, (F) Photoautotrophs in each sample. The first and second axes of Principal Co-ordinates Analysis illustrating Bray–Curtis distances between samples. **Fig. S4.** Effect of presence of *Culicoides* spp. on soil properties. Mean (A) Total Carbon, (B) Total Nitrogen, (C) Organic matter in soil containing *Culicoides* spp. and no *Culicoides* spp. The error bars are standard errors of the means. The value between bars indicates significant differences between with and without Culicoides spp. (*P* ≤ 0.05).

**Additional file 2: Table S1.** Alpha and beta diversity of bacteria, protista (overall protista, photoautotrophs, consumers, and parasites), fungi and metazoa in each sampling site (mean ± standard deviation). **Table S2.** Effects of habitat type, grazing type, and their interaction on abundances of microbial (bacterial, protistan, fungal and metazoan) phyla. **Table S3.** Relative abundance of major microbial (bacteria, protistan, fungi and metazoa) phyla in each sampling site (mean ± standard deviation). **Table S4.** Relative abundance of bacterial core communities in each sampling site (mean ± standard deviation). **Table S5.** Relative abundance of protistan core communities in each sampling site (mean ± standard deviation).

## Data Availability

The data that support the findings of this study are included in the article and supplementary materials. Also, raw sequence data are available at the National Center for Biotechnology Information Sequence Read Archive under the BioProject Number PRJNA862140.

## Abbreviations

KPBS: Konza prairie biological station
OM: Organic matter
OTU: Operational taxonomic unit
PCoA: Principal coordinates analysis
RDP: Ribosomal database project
rRNA: Ribosomal RNA
TC: Total carbon
TN: Total nitrogen
VSV: Vesicular stomatitis virus

## References

[1] Mullen GR, Murphree CS. Biting Midges (Ceratopogonidae). In: Medical and Veterinary Entomology. 3rd edition. Academic Press; 2019; 213–36.

[2] Gethmann J, Probst C, Conraths FJ (2020). Economic impact of a bluetongue serotype 8 epidemic in Germany. Front Vet Sci.

[3] Leder RR, Maas J, Lane VM, Evermann JF. Epidemiologic investigation of vesicular stomatitis in a dairy and its economic impact. Bov Pract. 1983; 45–49.

[4] Alderink FJ (1984). Vesicular stomatitis epidemic in Colorado: clinical observations and financial losses reported by dairymen. Prev Vet Med.

[5] Goodger WJ, Thurmond M, Nehay J, Mitchell J, Smith P (1985). Economic impact of an epizootic of bovine vesicular stomatitis in California. J Am Vet Med Assoc.

[6] Hayek AM, McCluskey BJ, Chavez GT, Salman MD (1998). Financial impact of the 1995 outbreak of vesicular stomatitis on 16 beef ranches in Colorado. J Am Vet Med Assoc.

[7] Blanton FS, Wirth WW. The sand flies (*Culicoides*) of Florida (Diptera: Ceratopogonidae). In: Arthropods of Florida and Neighboring Land Areas. Gainesville, FL: Florida Department of Agriculture and Consumer Services; 1979.

[8] Harrup LE, Purse BV, Golding N, Mellor PS, Carpenter S (2013). Larval development and emergence sites of farm associated *Culicoides* in the United Kingdom. Med Vet Entomol.

[9] Erram D, Blosser EM, Burkett-Cadena N (2019). Habitat associations of *Culicoides* species (Diptera: Ceratopogonidae) abundant on a commercial cervid farm in Florida, USA. Parasit Vectors.

[10] Battle FV, Turner EC (1972). Some nutritional and chemical properties of the larval habitats of certain species of *Culicoides* (Diptera: Ceratopogonidae)1. J Med Entomol.

[11] Schmidtmann ET, Bobian RJ, Belden RP (2000). Soil Chemistries define aquatic habitats with immature populations of the *Culicoides variipennis* complex (Diptera: Ceratopogonidae). J Med Entomol.

[12] Schmidtmann ET, Martin PAW (1992). Relationship between selected bacteria and the growth of immature house flies, *Musca domestica*, in an axenic test system. J Med Entomol.

[13] Lysyk TJ, Kalischuk-Tymensen L, Selinger LB, Lancaster RC, Wever L, Cheng K-J (1999). Rearing stable fly larvae (Diptera: Muscidae) on an egg yolk medium. J Med Entomol.

[14] Neupane S, Saski C, Nayduch D (2021). House fly larval grazing alters dairy cattle manure microbial communities. BMC Microbiol.

[15] Erram D, Burkett-Cadena N (2020). Laboratory rearing of *Culicoides stellifer* (Diptera: Ceratopogonidae), a suspected vector of orbiviruses in the United States. J Med Entomol.

[16] Parker MD, Akey DH, Lauerman LH (1977). Microbial flora associated with colonized and wild population of the biting gnat *Culicoides variipennis*. Entomol Exp Appl.

[17] Borkent A, Grogan WL (2009). Catalog of the new world biting midges north of Mexico (Diptera: Ceratopogonidae). Zootaxa.

[18] Wirth WW, Dyce AL, Peterson BV (1985). An atlas of wing photographs, with a summary of the numerical characters of the nearctic species of Culicoides (Diptera: Ceratopogonidae). Contrib Am Entomol Inst.

[19] Klindworth A, Pruesse E, Schweer T, Peplies J, Quast C, Horn M (2013). Evaluation of general 16S ribosomal RNA gene PCR primers for classical and next-generation sequencing-based diversity studies. Nucleic Acids Res.

[20] Choi J, Park JS (2020). Comparative analyses of the V4 and V9 regions of 18S rDNA for the extant eukaryotic community using the Illumina platform. Sci Rep.

[21] Schloss PD, Westcott SL, Ryabin T, Hall JR, Hartmann M, Hollister EB (2009). Introducing mothur: open-source, platform-independent, community-supported software for describing and comparing microbial communities. Appl Environ Microbiol.

[22] Yilmaz P, Parfrey LW, Yarza P, Gerken J, Pruesse E, Quast C (2014). The SILVA and “All-species Living Tree Project (LTP)” taxonomic frameworks. Nucleic Acids Res.

[23] Rognes T, Flouri T, Nichols B, Quince C, Mahé F (2016). VSEARCH: a versatile open-source tool for metagenomics. PeerJ.

[24] Wang Q, Garrity GM, Tiedje JM, Cole JR (2007). Naïve Bayesian classifier for rapid assignment of rRNA sequences into the new bacterial taxonomy. Appl Environ Microbiol.

[25] Cole JR, Wang Q, Cardenas E, Fish J, Chai B, Farris RJ (2008). The Ribosomal Database Project: improved alignments and new tools for rRNA analysis. Nucleic Acids Res.

[26] Guillou L, Bachar D, Audic S, Bass D, Berney C, Bittner L (2013). The Protist Ribosomal Reference database (PR2): a catalog of unicellular eukaryote small sub-unit rRNA sequences with curated taxonomy. Nucleic Acids Res.

[27] Adl SM, Bass D, Lane CE, Lukeš J, Schoch CL, Smirnov A (2019). Revisions to the classification, nomenclature, and diversity of eukaryotes. J Eukaryot Microbiol.

[28] Schulz G, Schneider D, Brinkmann N, Edy N, Daniel R, Polle A (2019). Changes in trophic groups of protists with conversion of rainforest into rubber and oil palm plantations. Front Microbiol.

[29] Nguyen B-AT, Chen Q-L, He J-Z, Hu H-W (2020). Oxytetracycline and ciprofloxacin exposure altered the composition of protistan consumers in an agricultural soil. Environ Sci Technol.

[30] R Development Core Team. R: A language and environment for statistical computing. 2019.

[31] Oksanen J, Guillaume BF, Friendly M, Kindt R, Legendre P, McGlinn D, et al. vegan: Community Ecology Package. 2.5–3. 2018.

[32] Bauer A, Cole CV, Black AL (1987). Soil property comparisons in virgin grasslands between grazed and non-grazed management systems. Soil Sci Soc Am J.

[33] Golluscio RA, Austin AT, García Martínez GC, Gonzalez-Polo M, Sala OE, Jackson RB (2009). Sheep grazing decreases organic carbon and nitrogen pools in the Patagonian steppe: combination of direct and indirect effects. Ecosyst.

[34] Möhlmann TWR, Bekendam AM, van Kemenade I, Wennergren U, Favia G, Takken W (2019). Latitudinal diversity of biting midge species within the *Obsoletus* group across three habitats in Europe. Med Vet Entomol.

[35] McGregor BL, Blackburn JK, Wisely SM, Burkett-Cadena ND (2021). *Culicoides* (Diptera: Ceratopogonidae) communities differ between a game preserve and nearby natural areas in northern Florida. J Med Entomol.

[36] Uslu U, Dik B (2010). Chemical characteristics of breeding sites of *Culicoides* species (Diptera: Ceratopogonidae). Vet Parasitol.

[37] Jangid K, Williams MA, Franzluebbers AJ, Blair JM, Coleman DC, Whitman WB (2010). Development of soil microbial communities during tallgrass prairie restoration. Soil Biol Biochem.

[38] Fierer N, Ladau J, Clemente JC, Leff JW, Owens SM, Pollard KS (2013). Reconstructing the microbial diversity and function of pre-agricultural tallgrass prairie soils in the United States. Science.

[39] Carson CM, Zeglin LH (2018). Long-term fire management history affects N-fertilization sensitivity, but not seasonality, of grassland soil microbial communities. Soil Biol Biochem.

[40] Uslu U, Dik B (2007). Description of breeding sites of *Culicoides* species (Diptera: Ceratopogonidae) in Turkey. Parasite.

[41] Zimmer J-Y, Haubruge E, Francis F, Bortels J, Simonon G, Losson B (2008). Breeding sites of bluetongue vectors in Northern Europe. Vet Rec.

[42] Pfannenstiel RS, Ruder MG (2015). Colonization of bison (Bison bison) wallows in a tallgrass prairie by *Culicoides* spp. (Diptera: Ceratopogonidae). J Vec Ecol..

[43] Barceló C, Estrada R, Lucientes J, Miranda MA (2020). A Mondrian matrix of seasonal patterns of *Culicoides nulliparous* and *parous* females at different latitudes in Spain. ResVet Sci.

[44] Lauber CL, Hamady M, Knight R, Fierer N (2009). Pyrosequencing-based assessment of soil pH as a predictor of soil bacterial community structure at the continental scale. Appl Environ Microbiol.

[45] Zhang K, Shi Y, Jing X, He J-S, Sun R, Yang Y (2016). Effects of short-term warming and altered precipitation on soil microbial communities in alpine grassland of the Tibetan Plateau. Front Microbiol.

[46] Clopton RE (1995). *Leidyana*
*migrator* n. sp. (Apicomplexa: Eugregarinida: Leidyanidae) from the Madagascar hissing cockroach, Gromphadorhina portentosa (Insecta: Blattodea). Invertebr Biol.

[47] Field SG, Schirp HJ, Michiels NK (2003). The influence of *Monocystis* sp. infection on growth and mating behaviour of the earthworm *Lumbricus*
*terrestris*. Can J Zool.

[48] Finlay BJ, Clarke KJ, Cowling AJ, Hindle RM, Rogerson A, Berninger U-G (1988). On the abundance and distribution of protozoa and their food in a productive freshwater pond. Eur J Protistol.

[49] Guhl BE, Finlay BJ, Schink B (1994). Seasonal development of hypolimnetic ciliate communities in a eutrophic pond. FEMS Microbiol Ecol.

[50] Chen X, Hou F, Wu Y, Cheng Y (2017). Bacterial and fungal community structures in Loess Plateau grasslands with different grazing intensities. Front Microbiol.

[51] Zhang H, Fu G (2021). Responses of plant, soil bacterial and fungal communities to grazing vary with pasture seasons and grassland types, northern Tibet. Land Degrad Dev.

[52] Eom AH, Wilson GWT, Hartnett DC (2001). Effects of ungulate grazers on arbuscular mycorrhizal symbiosis and fungal community structure in tallgrass prairie. Mycologia.

[53] Monchy S, Sanciu G, Jobard M, Rasconi S, Gerphagnon M, Chabé M (2011). Exploring and quantifying fungal diversity in freshwater lake ecosystems using rDNA cloning/sequencing and SSU tag pyrosequencing. Environ Microbiol.

[54] Rojas-Jimenez K, Wurzbacher C, Bourne EC, Chiuchiolo A, Priscu JC, Grossart H-P (2017). Early diverging lineages within Cryptomycota and Chytridiomycota dominate the fungal communities in ice-covered lakes of the McMurdo Dry Valleys. Antarctica Sci Rep.

[55] Jumpponen A, Jones KL, Blair J (2010). Vertical distribution of fungal communities in tallgrass prairie soil. Mycologia.

[56] Gleason FH, Marano AV, Johnson P, Martin WW (2010). Blastocladian parasites of invertebrates. Fungal Biol Rev.

[57] Porter TM, Martin W, James TY, Longcore JE, Gleason FH, Adler PH (2011). Molecular phylogeny of the Blastocladiomycota (Fungi) based on nuclear ribosomal DNA. Fungal Biol.

[58] Zinger L, Taberlet P, Schimann H, Bonin A, Boyer F, De Barba M (2019). Body size determines soil community assembly in a tropical forest. Mol Ecol.

[59] Yeates GW (2003). Nematodes as soil indicators: functional and biodiversity aspects. Biol Fert Soils.

[60] Timper P, Davies K, Gaugler R, Bilgrami AL (2004). Biotic interactions. Nematode Behaviour.

[61] Strayer DL. Freshwater Gastrotrichs. In: Reference Module in Earth Systems and Environmental Sciences. Elsevier; 2014.

[62] Johnson JS, Spakowicz DJ, Hong B-Y, Petersen LM, Demkowicz P, Chen L (2019). Evaluation of 16S rRNA gene sequencing for species and strain-level microbiome analysis. Nat Commun.

[63] Wu S, Xiong J, Yu Y (2015). Taxonomic resolutions based on 18S rRNA genes: a case study of subclass Copepoda. PLoS ONE.

[64] Hirakata Y, Hatamoto M, Oshiki M, Watari T, Kuroda K, Araki N (2019). Temporal variation of eukaryotic community structures in UASB reactor treating domestic sewage as revealed by 18S rRNA gene sequencing. Sci Rep.

