# Correction: Habitat type and host grazing regimen influence the soil microbial diversity and communities within potential biting midge larval habitats

**DOI:** 10.1186/s40793-023-00468-y

**Published:** 2023-02-28

**Authors:** Saraswoti Neupane, Travis Davis, Dana Nayduch, Bethany L. McGregor

**Affiliations:** 1 Department of Entomology, Kansas State University, Manhattan, KS 66506 USA; 2 Arthropod-Borne Animal Diseases Research Unit, USDA-ARS, Center for Grain and Animal Health Research, Manhattan, KS 66502 USA

**Correction to: Environmental Microbiome (2023) 18:5** 10.1186/s40793-022-00456-8

Following publication of the original article [1], the authors flagged that the following statement had been omitted in error from the Acknowledgements declaration: "The authors would like to thank Dr. Robert Pfannenstiel and Dr. Mark Ruder for their assistance with identifying the sites used in this study".

The published article has been updated to add the statement. The authors thank you for reading this correction and apologize for any inconvenience caused.
