# Supplementary material for: Habitat type and host grazing regimen influence the soil microbial diversity and communities within potential biting midge larval habitats
Source: Environ Microbiome. 2023 Jan 19;18:5. doi: 10.1186/s40793-022-00456-8 (PMC9854200; doi:10.1186/s40793-022-00456-8)
Supplement: Supplementary file 1 — Additional file 1: Fig. S1. Trophic groups of soil protistan communities. Mean relative abundance of (A) Consumers, (B) Parasites, (C) Photoautotrophs, (D) Photoautotrophs/Consumers, (E) Consumers/Plant Pathogens, (F) Symbionts, and (G) Unassigned groups in disturbed (bison- and cattle-grazed) and undisturbed (non-grazed) pond and spring habitats. The error bars are standard errors of the means. Fig. S2. Soil protistan trophic group communities. Relative abundances protistan phyla (A) Consumers, (B) Parasites, (C) Photoautotrophs in disturbed (bison- and cattle-grazed) and undisturbed (non-grazed) pond and spring habitats. Fig. S3. Soil protistan trophic group diversity and community composition. Shannon diversity index of (A) Consumers, (B) Parasites, (C) Photoautotrophs in disturbed (bison- and cattle-grazed) and undisturbed (non-grazed) pond and spring habitats. The error bars are standard errors of means. The different letters indicate the significant differences between sampling sites (P ≤ 0.05). Protistan trophic groups community composition (D) Consumers, (E) Parasites, (F) Photoautotrophs in each sample. The first and second axes of Principal Co-ordinates Analysis illustrating Bray–Curtis distances between samples. Fig. S4. Effect of presence of Culicoides spp. on soil properties. Mean (A) Total Carbon, (B) Total Nitrogen, (C) Organic matter in soil containing Culicoides spp. and no Culicoides spp. The error bars are standard errors of the means. The value between bars indicates significant differences between with and without Culicoides spp. (P ≤ 0.05). [file 40793_2022_456_MOESM1_ESM.pdf]

Supplementary Materials

Habitat type and host grazing regimen influence the soil microbial diversity and communities within potential biting midge larval habitats

Saraswoti Neupane[1*], Travis Davis[2], Dana Nayduch[2], Bethany L. McGregor[2]

[1]Department of Entomology, Kansas State University, Manhattan, KS 66506, USA[2]USDA-ARS, Center for Grain and Animal Health Research, Arthropod-Borne Animal Diseases Research Unit, Manhattan, KS 66502, USA

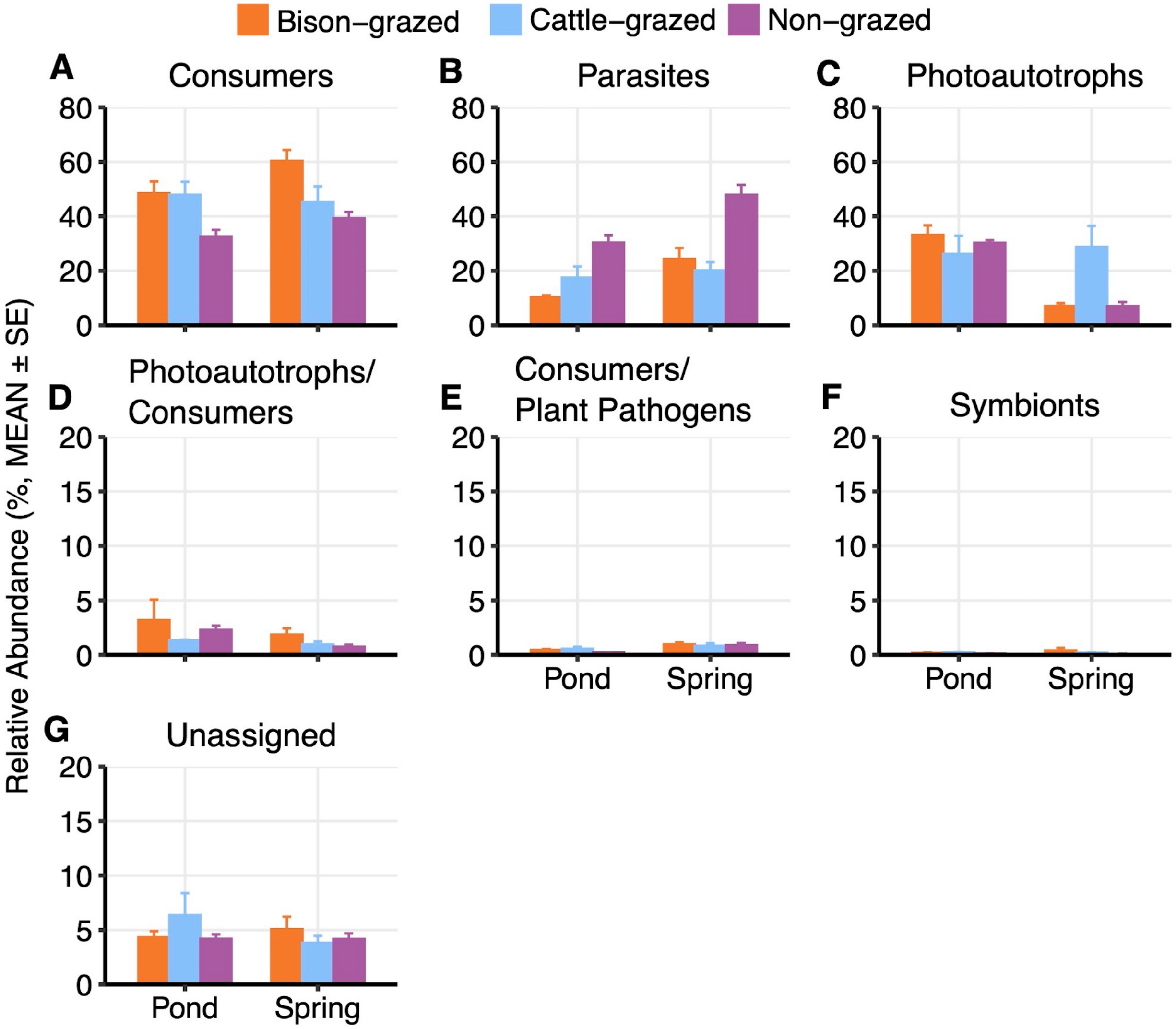

Fig. S1: Trophic groups of soil protistan communities. Mean relative abundance of (A) Consumers, (B) Parasites, (C) Photoautotrophs, (D) Photoautotrophs/Consumers, (E) Consumers/Plant Pathogens, (F) Symbionts, and (G) Unassigned groups in disturbed (bison- and cattle-grazed) and undisturbed (non-grazed) pond and spring habitats. The error bars are standard errors of the means.

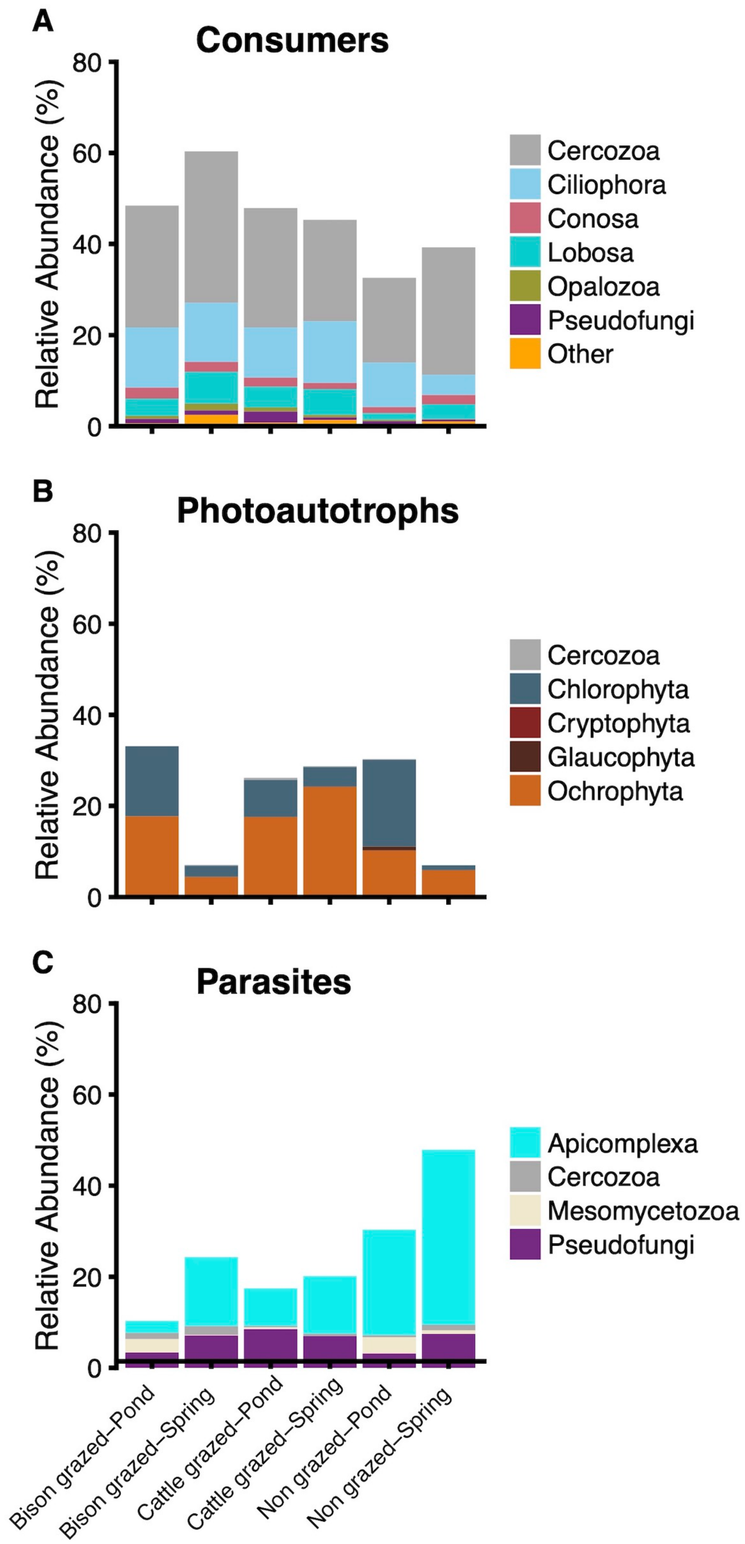

Fig. S2: Soil protistan trophic group communities. Relative abundances protistan phyla (A) Consumers, (B) Parasites, (C) Photoautotrophs in disturbed (bison- and cattle-grazed) and undisturbed (non-grazed) pond and spring habitats.

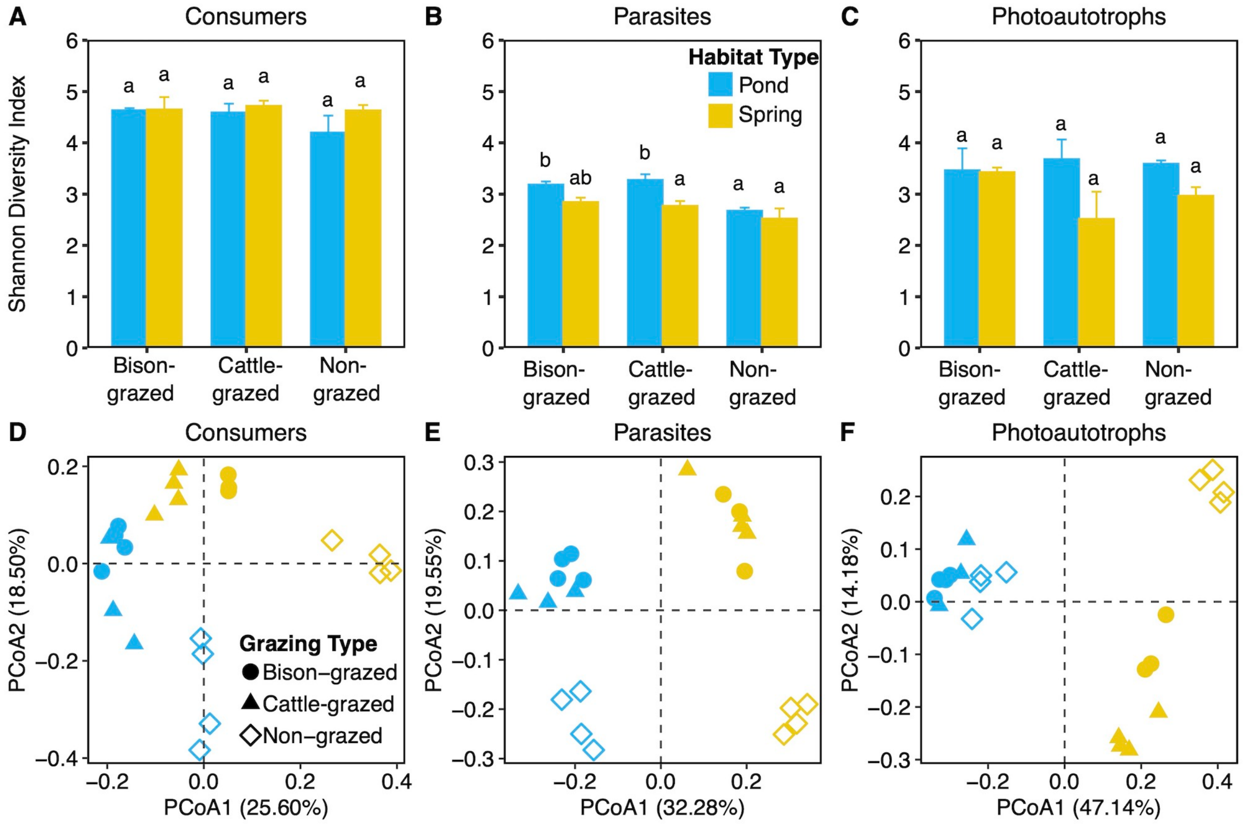

Fig. S3: Soil protistan trophic group diversity and community composition. Shannon diversity index of (A) Consumers, (B) Parasites, (C) Photoautotrophs in disturbed (bison- and cattle-grazed) and undisturbed (non-grazed) pond and spring habitats. The error bars are standard errors of means. The different letters indicate the significant differences between sampling sites ($P \leq 0.05$). Protistan trophic groups community composition (D) Consumers, (E) Parasites, (F) Photoautotrophs in each sample. The first and second axes of Principal Co-ordinates Analysis illustrating Bray-Curtis distances between samples.

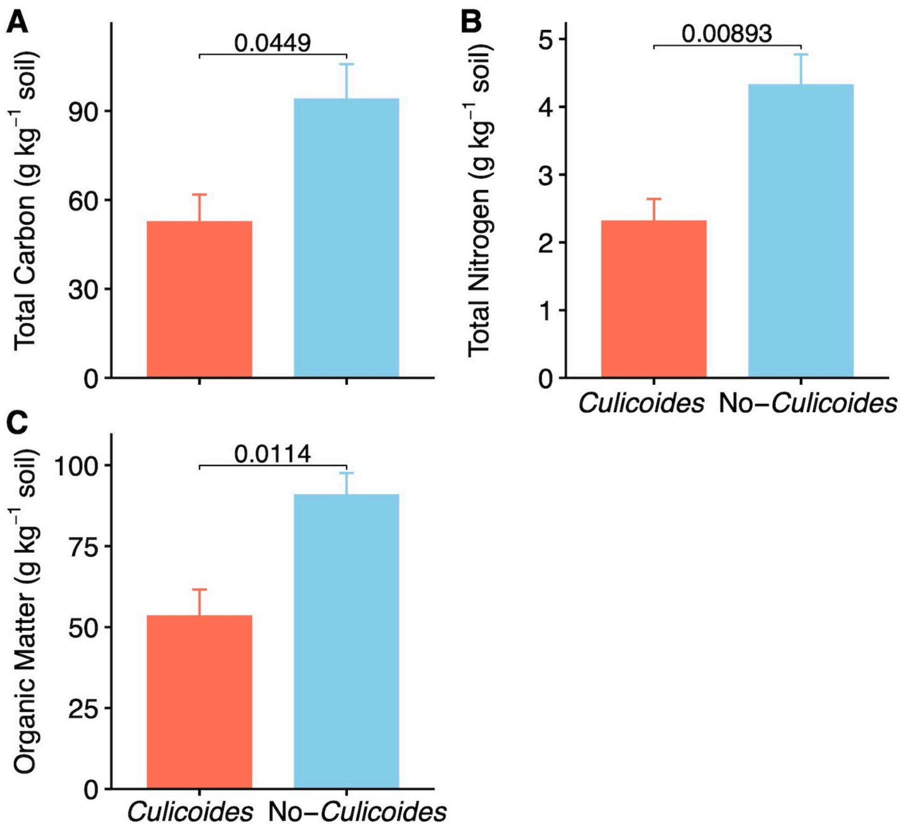

Fig. S4: Effect of presence of *Culicoides* spp. on soil properties. Mean (A) Total Carbon, (B) Total Nitrogen, (C) Organic matter in soil containing *Culicoides* spp. and no *Culicoides* spp. The error bars are standard errors of the means. The value between bars indicates significant differences between with and without *Culicoides* spp. ($P \leq 0.05$).
