# Supplementary material for: Habitat type and host grazing regimen influence the soil microbial diversity and communities within potential biting midge larval habitats
Source: Environ Microbiome. 2023 Jan 19;18:5. doi: 10.1186/s40793-022-00456-8 (PMC9854200; doi:10.1186/s40793-022-00456-8)
Supplement: Supplementary file 2 — Additional file 2: Table S1. Alpha and beta diversity of bacteria, protista (overall protista, photoautotrophs, consumers, and parasites), fungi and metazoa in each sampling site (mean ± standard deviation). Table S2. Effects of habitat type, grazing type, and their interaction on abundances of microbial (bacterial, protistan, fungal and metazoan) phyla. Table S3. Relative abundance of major microbial (bacteria, protistan, fungi and metazoa) phyla in each sampling site (mean ± standard deviation). Table S4. Relative abundance of bacterial core communities in each sampling site (mean ± standard deviation). Table S5. Relative abundance of protistan core communities in each sampling site (mean ± standard deviation). [file 40793_2022_456_MOESM2_ESM.pdf]

Supplementary Materials

Habitat type and host grazing regimen influence the soil microbial diversity and communities within potential biting midge larval habitats

Saraswoti Neupane[1*], Travis Davis[2], Dana Nayduch[2], Bethany L. McGregor[2]

[1]Department of Entomology, Kansas State University, Manhattan, KS 66506, USA[2]USDA-ARS, Center for Grain and Animal Health Research, Arthropod-Borne Animal Diseases Research Unit, Manhattan, KS 66502, USA

Table S1: Alpha and beta diversity of bacteria, protista (overall protista, photoautotrophs, consumers, and parasites), fungi and metazoa in each sampling site (mean ± standard deviation).

Kingdom	Trophic Level	Diversity	Bison-grazed		Cattle-grazed		Non-grazed	
			Pond	Spring	Pond	Spring	Pond	Spring
Bacteria		Richness	2484.75 ± 177.42 a	2381 ± 135.4 a	2310.33 ± 209.28 a	2651 ± 327.2 a	2615.25 ± 201.15 a	2798.5 ± 238.95 a
		Evenness	0.87 ± 0 bc	0.89 ± 0.01 bc	0.84 ± 0.01 a	0.88 ± 0.01 bc	0.86 ± 0.01 b	0.88 ± 0.01 c
		Beta diversity	0.54 ± 0.01 ab	0.52 ± 0.01 ab	0.56 ± 0.02 b	0.51 ± 0.02 a	0.56 ± 0.03 b	0.55 ± 0.02 b
Protista	Overall	Richness	778.5 ± 46.84 b	650 ± 57.17 ab	625.67 ± 178.18 ab	650.5 ± 115.51 ab	627.75 ± 109.37 ab	488.5 ± 44.68 a
		Evenness	0.76 ± 0.07 a	0.78 ± 0.04 a	0.81 ± 0.07 a	0.72 ± 0.08 a	0.75 ± 0.03 a	0.72 ± 0.06 a
		Beta diversity	0.6 ± 0.01 a	0.63 ± 0.01 ab	0.63 ± 0.03 ab	0.61 ± 0.02 a	0.63 ± 0.03 ab	0.67 ± 0.02 b
	Photoautotrophs	Richness	142.5 ± 4.36 c	66.67 ± 7.57 ab	124 ± 23.39 c	82.25 ± 9.64 b	135.5 ± 12.18 c	44 ± 5.48 a
		Evenness	0.7 ± 0.17 a	0.82 ± 0.04 a	0.77 ± 0.15 a	0.57 ± 0.23 a	0.73 ± 0.03 a	0.78 ± 0.07 a
		Beta diversity	0.6 ± 0.01 a	0.67 ± 0.01 c	0.63 ± 0.02 ab	0.64 ± 0.02 bc	0.59 ± 0.02 a	0.71 ± 0.01 d
	Consumers	Richness	440.75 ± 31.95 a	409 ± 28.58 a	328.33 ± 107.87 a	397.25 ± 77.49 a	311.25 ± 75.29 a	299 ± 28.12 a
		Evenness	0.76 ± 0.01 a	0.77 ± 0.06 a	0.8 ± 0.06 a	0.79 ± 0.02 a	0.73 ± 0.1 a	0.81 ± 0.03 a
		Beta diversity	0.57 ± 0 a	0.58 ± 0.01 ab	0.61 ± 0.04 ab	0.57 ± 0.02 a	0.61 ± 0.04 ab	0.64 ± 0.03 b
	Parasites	Richness	71.75 ± 2.75 a	72.33 ± 17.79 a	70.67 ± 14.01 a	75.5 ± 11.7 a	77.25 ± 2.22 a	78.5 ± 5.07 a
		Evenness	0.75 ± 0.02 bc	0.67 ± 0.03 abc	0.77 ± 0.01 c	0.64 ± 0.06 ab	0.62 ± 0.03 a	0.58 ± 0.09 a
		Beta diversity	0.63 ± 0.01 a	0.66 ± 0.03 ab	0.66 ± 0.01 ab	0.64 ± 0.02 ab	0.64 ± 0.01 ab	0.67 ± 0.01 b
Fungi		Richness	137.75 ± 8.96 b	124.33 ± 13.65 ab	107 ± 30.45 ab	114 ± 18.57 ab	102.25 ± 10.53 ab	99.5 ± 9.11 a
		Evenness	0.77 ± 0.03 a	0.78 ± 0.1 a	0.71 ± 0.07 a	0.71 ± 0.06 a	0.79 ± 0.03 a	0.71 ± 0.07 a
		Beta diversity	0.65 ± 0.01 a	0.67 ± 0 ab	0.66 ± 0.02 ab	0.66 ± 0.01 ab	0.65 ± 0.01 ab	0.68 ± 0.01 b
Metazoa		Richness	35 ± 6.83 ab	29.67 ± 2.08 a	40 ± 10.15 ab	30.25 ± 8.14 a	47.5 ± 4.8 bc	58.25 ± 7.8 c
		Evenness	0.66 ± 0.09 a	0.6 ± 0.11 a	0.68 ± 0.09 a	0.59 ± 0.1 a	0.57 ± 0.06 a	0.6 ± 0.11 a
		Beta diversity	0.69 ± 0.02 ab	0.8 ± 0.03 c	0.75 ± 0.02 bc	0.72 ± 0.04 ab	0.68 ± 0.01 a	0.73 ± 0.02 ab

Richness = species richness, Evenness = Pielou's evenness.
The different letters after standard deviation indicate significant differences between sampling sites/or potential midge larval habitats ($P \leq 0.05$).

Table S2: Effects of habitat type, grazing type, and their interaction on abundances of microbial (bacterial, protistan, fungal and metazoan) phyla.

Kingdom	Phyla	Habitat		Grazing		Habitat:Grazing	
		$F_{(1,16)*}$	*P*-value	$F_{(2,16)*}$	*P*-value	$F_{(2,16)*}$	*P*-value
**Bacteria**	Acidobacteria	6.09	**0.0253**	27.73	**<0.0001**	3.87	**0.0426**
	Actinobacteria	10.98	**0.0044**	23.77	**<0.0001**	9.66	**0.0018**
	Bacteroidetes	5.53	**0.0318**	12.22	**0.0006**	0.53	0.601
	Chloroflexi	6.5	**0.0214**	5.31	**0.017**	30.16	**<0.0001**
	Firmicutes	6.09	**0.0252**	24.59	**<0.0001**	5.85	**0.0124**
	Planctomycetes	3.72	0.0717	215.83	**<0.0001**	3.65	**0.0494**
	Proteobacteria	3.89	**0.066**	22.21	**<0.0001**	9.41	**0.002**
	Verrucomicrobia	11.13	**0.0042**	2.62	0.104	5.82	**0.0126**
	Other_Bacteria	0.32	0.579	13.71	**0.0003**	0.34	0.7191
**Protista**	Apicomplexa	18.19	**0.0006**	31.59	**<0.0001**	1.55	0.2434
	Cercozoa	1.37	0.2594	2.87	0.0858	2.78	0.0918
	Chlorophyta	89.53	**<0.0001**	2.19	0.1443	10.44	**0.0013**
	Ciliophora	0.63	0.4374	5.87	**0.0123**	2.1	0.1547
	Conosa	0.05	0.8208	1.74	0.2079	1.52	0.2478
	Lobosa	11.75	**0.0035**	11.06	**0.001**	1	0.3898
	Ochrophyta	0.71	0.4112	2.63	0.1031	1.48	0.2567
	Other_Protista	0.37	0.5531	0.38	0.6919	1.91	0.1797
**Fugni**	Ascomycota	29.01	**<0.0001**	4.26	**0.0328**	3.62	0.0506
	Basidiomycota	3.83	0.068	0.46	0.642	0.08	0.9223
	Blastocladiomycota	0.24	0.6283	10.03	**0.0015**	0.14	0.8715
	Chytridiomycota	1.84	0.194	1.25	0.3131	0.31	0.7354
	Cryptomycota	0.62	0.4419	12.04	**0.0006**	0.92	0.419
	Other_Fungi	10.47	**0.0052**	8.56	**0.003**	0.99	0.3947
**Metazoa**	Annelida	5.12	**0.0379**	2.4	0.1228	1.02	0.3838
	Arthropoda	2.57	0.1286	2.21	0.1416	0.71	0.5057

Gastrotricha	9.46	**0.0072**	0.67	0.525	1.09	0.3612
Nematoda	10.76	**0.0047**	3.01	0.0775	0.47	0.6306
Platyhelminthes	0.17	0.6839	6.29	**0.0097**	17.73	**<0.0001**
Rotifera	4.08	0.0605	0.48	0.6279	1.31	0.2968
Other_Metazoa	0.27	0.6111	0.19	0.8269	0.95	0.4088

* Degree of freedom. Significant *P*-values are in bold ($P \leq 0.05$).

Table S3: Relative abundance of major microbial (bacteria, protistan, fungi and metazoa) phyla in each sampling site (mean ± standard deviation).

Kingdom	Phyla	Bison-grazed		Cow-grazed		Non-grazed	
		Pond	Spring	Pond	Spring	Pond	Spring
**Bacteria**	Acidobacteria	14.56 ± 0.62 c	14.11 ± 0.6 c	7.41 ± 1.42 a	10.19 ± 1.65 ab	9.43 ± 0.88 a	12.89 ± 2.3 bc
	Actinobacteria	23.46 ± 1.88 cd	24.09 ± 1.65 d	17.64 ± 0.97 bc	18.04 ± 2.47 b	10.91 ± 2 a	20.73 ± 3.76 bcd
	Bacteroidetes	3.61 ± 0.47 ab	2.31 ± 0.81 a	5.28 ± 0.98 b	4.79 ± 0.74 b	5.41 ± 0.72 b	4.1 ± 1.24 ab
	Chloroflexi	7.35 ± 1.27 bc	7.25 ± 0.52 bc	6.33 ± 2.79 b	9.7 ± 1.2 c	9.63 ± 0.79 c	2.67 ± 0.3 a
	Firmicutes	8.49 ± 1.5 a	4.26 ± 0.62 a	19.46 ± 4.02 b	10.77 ± 4.75 a	4.86 ± 0.51 a	5.97 ± 2.18 a
	Planctomycetes	2.52 ± 0.13 a	3.53 ± 0.09 b	3.03 ± 0.61 ab	3.04 ± 0.48 ab	6.69 ± 0.59 c	6.7 ± 0.11 c
	Proteobacteria	27.71 ± 0.88 a	31.29 ± 2.37 ab	26.3 ± 2.48 a	33.53 ± 2.55 bc	39.07 ± 1.54 c	35.3 ± 3.99 bc
	Verrucomicrobia	3.5 ± 1.06 a	3.99 ± 0.41 a	8.41 ± 3.98 b	2.96 ± 0.44 a	6.11 ± 1.3 ab	3.65 ± 0.42 a
	Other_Bacteria	8.8 ± 0.57 bc	9.16 ± 0.32 c	6.13 ± 1.53 a	6.98 ± 1.11 ab	7.85 ± 0.21 abc	7.97 ± 0.82 abc
**Protista**	Apicomplexa	2.67 ± 0.44 a	15.19 ± 6.96 ab	8.29 ± 2.11 ab	12.7 ± 5.41 ab	23.23 ± 4.58 b	38.47 ± 10.58 c
	Cercozoa	32.2 ± 6.94 ab	40.16 ± 3.54 b	32.69 ± 9.88 ab	26.53 ± 8.77 ab	23.14 ± 6.89 a	32.65 ± 2.76 ab
	Chlorophyta	15.39 ± 5.03 bc	2.47 ± 1.75 a	8.16 ± 1.85 ab	4.34 ± 3.08 a	19.05 ± 2.98 c	1.05 ± 0.38 a
	Ciliophora	13.21 ± 5.08 b	12.99 ± 5.99 ab	11.02 ± 0.79 ab	13.54 ± 3.93 b	9.75 ± 2.47 ab	4.42 ± 1.96 a
	Conosa	2.38 ± 1.19 a	2.16 ± 0.56 a	1.95 ± 0.94 a	1.31 ± 0.52 a	1.32 ± 0.54 a	2.03 ± 0.61 a
	Lobosa	3.83 ± 0.9 abc	7.01 ± 1.83 c	4.6 ± 2.37 abc	5.74 ± 1.57 bc	1.48 ± 0.66 a	3.27 ± 0.55 ab
	Ochrophyta	20.9 ± 15.46 a	6.34 ± 1.61 a	19.07 ± 12.7 a	25.28 ± 16.96 a	12.7 ± 5.49 a	6.77 ± 2.5 a
	Other_Protista	9.4 ± 1.67 a	13.67 ± 0.76 abc	14.21 ± 4.84 ab	10.56 ± 1.23 ab	9.33 ± 2.09 bc	11.33 ± 7.43 c
**Fungi**	Ascomycota	1.77 ± 1.39 ab	16.95 ± 9.05 c	1.3 ± 0.32 ab	5.21 ± 3.8 ab	1.21 ± 0.85 a	10.54 ± 2.92 bc
	Basidiomycota	2.46 ± 1.67 a	8.36 ± 2.72 a	4.73 ± 5.48 a	9.78 ± 11.32 a	2.66 ± 2.12 a	6.14 ± 4.97 a
	Blastocladiomycota	11.83 ± 5.59 a	13.31 ± 3.29 a	8.83 ± 4.32 a	11.78 ± 10.63 a	0.66 ± 0.27 a	0.57 ± 0.42 a
	Chytridiomycota	28.25 ± 13.17 a	20.18 ± 5.7 a	26.29 ± 7.62 a	20.7 ± 5.77 a	30.42 ± 8.33 a	29.16 ± 6.49 a
	Cryptomycota	23.05 ± 6 ab	12.22 ± 5.99 a	27.36 ± 4.26 ab	26.99 ± 10.25ab	39.48 ± 6.79 b	39.15 ± 12.29 b
	Other_Fungi	32.63 ± 6.26 b	28.99 ± 4.14 b	31.49 ± 8.14 b	25.54 ± 4.38 ab	25.57 ± 3.6 ab	14.44 ± 4.76 a
**Metazoa**	Annelida	0.52 ± 0.56 a	0.13 ± 0.22 a	3.42 ± 2.92 a	0.72 ± 0.45 a	1.8 ± 2.4 a	0.08 ± 0.06 a

Arthropoda	29.45 ± 21.62 a	20.34 ± 34.34 a	16.38 ± 7.77 a	13.73 ± 15.34 a	53.8 ± 19.79 a	25.15 ± 25.83 a
Gastrotricha	9.75 ± 4.33 a	0.47 ± 0.62 a	11.92 ± 10.42 a	0.24 ± 0.44 a	10.01 ± 8.6 a	6.95 ± 4.21 a
Nematoda	42.35 ± 8.38 ab	54.61 ± 27.81ab	43.45 ± 24.58ab	71.71 ± 10.88 b	24.14 ± 13.24 a	50.08 ± 13.41ab
Platyhelminthes	3.98 ± 4.28 a	22.01 ± 7.81 b	9.89 ± 5.33 a	0.72 ± 1.29 a	6.24 ± 2.81 a	2.6 ± 4.51 a
Rotifera	5.98 ± 3.24 a	1.11 ± 0.42 a	6.05 ± 5.02 a	2.8 ± 1.25 a	2.93 ± 2.56 a	2.87 ± 3.19 a
Other_Metazoa	7.98 ± 15.42 a	1.34 ± 2.14 a	8.9 ± 14.83 a	10.08 ± 10.49 a	1.09 ± 1.47 a	12.27 ± 18.34 a

The different letters after standard deviation indicate significant differences between sampling sites (potential midge larval habitats) ($P \leq 0.05$).

Table S4: Relative abundance of bacterial core communities in each sampling site (mean ± standard deviation).

Taxon	Pond			Spring		
	Bison	Cow	Non-grazing	Bison	Cow	Non-grazing
Bacteria_unclassified	6.32 ± 0.67	3.96 ± 0.84	4.93 ± 0.22	7.48 ± 0.38	4.95 ± 0.98	6.15 ± 0.91
Gp6_unclassified	3.97 ± 0.41	2.05 ± 0.6	2.18 ± 0.42	5.53 ± 0.2	3.62 ± 0.85	4.93 ± 1.05
Rhizobiales_unclassified	4.14 ± 0.42	2.89 ± 0.54	3.13 ± 0.45	3.76 ± 0.35	2.48 ± 0.23	4.95 ± 0.5
Anaerolineaceae_unclassified	4.08 ± 0.94	3.49 ± 1.82	3.19 ± 0.39	3.99 ± 0.31	5.11 ± 0.41	1.02 ± 0.26
Betaproteobacteria_unclassified	1.93 ± 0.07	1.59 ± 0.49	3.84 ± 0.54	3.39 ± 0.2	2.83 ± 0.64	3.81 ± 0.69
Gp16_unclassified	4.3 ± 0.23	2.41 ± 0.46	2.76 ± 0.18	3.01 ± 0.28	2.17 ± 0.38	2.03 ± 0.36
Gaiella	3.28 ± 1.16	0.63 ± 0.12	0.63 ± 0.21	4.23 ± 0.59	2.05 ± 0.4	4.57 ± 1.24
Actinobacteria_unclassified	2.52 ± 0.28	1.84 ± 0.7	1.88 ± 0.2	3.63 ± 0.31	1.85 ± 0.31	2.9 ± 0.52
Thermoleophilia_unclassified	1.72 ± 0.12	1.19 ± 0.16	0.97 ± 0.14	4.51 ± 0.75	1.33 ± 0.28	2.21 ± 0.99
Bacillaceae_1_unclassified	1.63 ± 0.25	4.4 ± 0.79	0.4 ± 0.07	0.99 ± 0.23	1.69 ± 0.6	1.73 ± 0.42
Chloroflexi_unclassified	1.93 ± 0.27	1.8 ± 0.68	1.72 ± 0.3	1.72 ± 0.17	2.07 ± 0.6	0.53 ± 0.05
Luteolibacter	0.87 ± 0.96	4.79 ± 2.84	2.5 ± 0.74	0.14 ± 0.06	0.6 ± 0.31	0.62 ± 0.36
Steroidobacteraceae_unclassified	0.81 ± 0.12	1.48 ± 0.29	2.77 ± 0.46	1.32 ± 0.1	1.67 ± 0.23	0.8 ± 0.16
Bacteroidetes_unclassified	0.73 ± 0.12	1.83 ± 0.27	2.48 ± 0.48	0.69 ± 0.23	1.62 ± 0.26	0.82 ± 0.25
Subdivision3_unclassified	1.53 ± 0.17	1.47 ± 0.33	1.89 ± 0.44	1.31 ± 0.13	1.19 ± 0.06	0.94 ± 0.06
Intrasporangiaceae_unclassified	2.63 ± 0.37	1.49 ± 0.17	0.57 ± 0.15	1.33 ± 0.2	1.88 ± 0.33	0.3 ± 0.09
Acidimicrobiales_unclassified	1.33 ± 0.16	0.83 ± 0.11	0.9 ± 0.15	1.77 ± 0.23	0.93 ± 0.1	1.29 ± 0.21
Clostridium_sensu_stricto	1.3 ± 0.35	2.39 ± 0.46	1.16 ± 0.4	0.47 ± 0.1	1.12 ± 0.38	0.48 ± 0.19
Caldilineaceae_unclassified	0.41 ± 0.09	0.54 ± 0.02	2.98 ± 0.19	0.69 ± 0.06	1.13 ± 0.23	0.56 ± 0.13
Comamonadaceae_unclassified	0.98 ± 0.33	1.08 ± 0.7	1.35 ± 0.5	0.89 ± 0.24	1.3 ± 0.17	1.05 ± 0.48
Gp17_unclassified	0.7 ± 0.13	0.68 ± 0.07	1.36 ± 0.16	0.99 ± 0.11	0.95 ± 0.24	1.73 ± 0.34
Rhodobacteraceae_unclassified	0.61 ± 0.15	1.26 ± 0.54	2.47 ± 0.65	0.28 ± 0.14	0.57 ± 0.25	1.01 ± 0.29
Sphingomonas	2.3 ± 0.31	0.77 ± 0.21	0.36 ± 0.2	0.76 ± 0.06	1.49 ± 0.26	0.82 ± 0.35
Solirubrobacterales_unclassified	1.79 ± 0.38	0.98 ± 0.37	0.52 ± 0.16	1.03 ± 0.27	0.78 ± 0.28	1.25 ± 0.49

Pirellulales_unclassified	0.46 ± 0.06	1.13 ± 0.31	1.52 ± 0.13	0.81 ± 0.12	0.64 ± 0.13	1.47 ± 0.05
Deltaproteobacteria_unclassified	0.26 ± 0.07	1.03 ± 0.15	1.31 ± 0.06	1.08 ± 0.06	0.66 ± 0.24	1.42 ± 0.32
Coriobacteriia_unclassified	1.1 ± 0.17	3 ± 0.57	0.7 ± 0.07	0.38 ± 0.02	0.75 ± 0.24	0.2 ± 0.18
Burkholderiales_unclassified	0.53 ± 0.13	0.81 ± 0.16	1.28 ± 0.09	0.88 ± 0.04	1.09 ± 0.15	0.71 ± 0.14
Gammaproteobacteria_unclassified	0.45 ± 0.11	0.42 ± 0.12	0.82 ± 0.04	1.06 ± 0.22	0.65 ± 0.2	1.69 ± 0.21
Hyphomicrobium	0.81 ± 0.11	0.82 ± 0.19	1.31 ± 0.18	0.36 ± 0.06	0.48 ± 0.05	1.11 ± 0.21
Planococcaceae_unclassified	0.72 ± 0.16	3.35 ± 1.29	0.06 ± 0.02	0.19 ± 0.06	1.02 ± 0.26	0.15 ± 0.05
Chitinophagaceae_unclassified	0.79 ± 0.1	0.85 ± 0.09	1.31 ± 0.14	0.32 ± 0.11	0.62 ± 0.1	0.85 ± 0.14
Gp3_unclassified	1.44 ± 0.15	0.37 ± 0.17	0.56 ± 0.1	0.93 ± 0.18	0.72 ± 0.13	0.49 ± 0.11
Ilumatobacteraceae_unclassified	0.7 ± 0.13	0.26 ± 0.08	0.45 ± 0.08	1.01 ± 0.1	0.64 ± 0.17	1.27 ± 0.16
Methylocystis	0.79 ± 0.18	1.38 ± 0.5	1.16 ± 0.25	0.25 ± 0.02	0.38 ± 0.06	0.35 ± 0.19
Myxococcales_unclassified	0.61 ± 0.11	0.81 ± 0.29	0.32 ± 0.05	1.1 ± 0.34	0.96 ± 0.18	0.52 ± 0.09
Nocardioides	1.07 ± 0.16	0.45 ± 0.11	0.39 ± 0.2	0.69 ± 0.21	0.83 ± 0.2	0.62 ± 0.19
Anaeromyxobacter	2 ± 0.33	0.49 ± 0.16	0.15 ± 0.07	0.46 ± 0.03	0.58 ± 0.34	0.17 ± 0.05
Marmoricola	0.66 ± 0.12	0.81 ± 0.25	0.67 ± 0.35	0.36 ± 0.19	0.96 ± 0.19	0.19 ± 0.24
Rhodospirillales_unclassified	0.24 ± 0.07	0.06 ± 0.01	0.38 ± 0.11	1.16 ± 0.22	0.48 ± 0.1	1.29 ± 0.2
Hyphomicrobiaceae_unclassified	0.38 ± 0.09	0.24 ± 0.05	0.44 ± 0.16	1 ± 0.1	0.5 ± 0.05	0.99 ± 0.25
Spartobacteria_unclassified	0.47 ± 0.33	0.49 ± 0.25	0.42 ± 0.14	1.58 ± 0.39	0.26 ± 0.1	0.55 ± 0.09
Pseudarthrobacter	0.9 ± 0.16	0.68 ± 0.23	0.18 ± 0.09	1.15 ± 0.74	0.38 ± 0.06	0.32 ± 0.14
Isosphaeraceae_unclassified	0.33 ± 0.06	0.21 ± 0.06	1.54 ± 0.36	0.15 ± 0.03	0.24 ± 0.05	0.17 ± 0.04
Iamiaceae_unclassified	0.7 ± 0.22	0.47 ± 0.09	0.24 ± 0.13	0.51 ± 0.07	0.48 ± 0.09	0.5 ± 0.08
Micromonosporaceae_unclassified	0.69 ± 0.12	0.88 ± 0.24	0.14 ± 0.04	0.35 ± 0.09	0.42 ± 0.08	0.44 ± 0.06
Novosphingobium	0.23 ± 0.09	0.32 ± 0.13	0.05 ± 0.02	0.34 ± 0.15	0.54 ± 0.2	1.18 ± 0.65
Bradyrhizobiaceae_unclassified	0.38 ± 0.08	0.37 ± 0.14	0.48 ± 0.12	0.59 ± 0.06	0.46 ± 0.06	0.51 ± 0.03
Romboutsia	0.39 ± 0.14	1.94 ± 0.55	0.11 ± 0.03	0.11 ± 0.04	0.5 ± 0.24	0.03 ± 0.01
Microvirga	0.59 ± 0.15	0.43 ± 0.12	0.13 ± 0.05	0.22 ± 0.03	0.7 ± 0.1	0.54 ± 0.36
Bacteroidales_unclassified	0.43 ± 0.07	0.87 ± 0.33	0.46 ± 0.25	0.18 ± 0.07	0.76 ± 0.36	0.08 ± 0.07
Bradyrhizobium	0.69 ± 0.44	0.4 ± 0.09	0.22 ± 0.12	0.52 ± 0.08	0.48 ± 0.06	0.45 ± 0.05
Methylococcaceae_unclassified	0.07 ± 0.03	0.14 ± 0.09	1.74 ± 0.52	0.08 ± 0.02	0.15 ± 0.08	0.09 ± 0.05

Ruminococcaceae_unclassified	0.47 ± 0.09	0.78 ± 0.12	0.38 ± 0.01	0.21 ± 0.06	0.41 ± 0.23	0.32 ± 0.27
Verrucomicrobiaceae_unclassified	0.21 ± 0.15	1.22 ± 0.64	0.45 ± 0.13	0.12 ± 0.01	0.19 ± 0.01	0.42 ± 0.08
Desulfobacteraceae_unclassified	0.13 ± 0.03	0.31 ± 0.12	0.87 ± 0.29	0.44 ± 0.06	0.5 ± 0.26	0.22 ± 0.02
Geobacter	0.37 ± 0.13	0.47 ± 0.42	0.64 ± 0.13	0.48 ± 0.27	0.3 ± 0.13	0.23 ± 0.08
Alphaproteobacteria_unclassified	0.17 ± 0.02	0.18 ± 0.05	0.6 ± 0.08	0.5 ± 0.05	0.19 ± 0.03	0.67 ± 0.11
candidate_division_WPS.1	0.36 ± 0.07	0.15 ± 0.04	0.36 ± 0.07	0.29 ± 0.05	0.43 ± 0.09	0.58 ± 0.18
Kofleria	0.42 ± 0.08	0.42 ± 0.22	0.24 ± 0.08	0.57 ± 0.11	0.33 ± 0.05	0.36 ± 0.05
Gemmataceae_unclassified	0.17 ± 0.07	0.1 ± 0.04	0.43 ± 0.09	0.4 ± 0.04	0.23 ± 0.05	0.75 ± 0.04
Gp10_unclassified	0.34 ± 0.07	0.11 ± 0.07	0.48 ± 0.31	0.42 ± 0.05	0.27 ± 0.13	0.44 ± 0.11
Gp4_unclassified	0.5 ± 0.15	0.03 ± 0.01	0.04 ± 0.04	0.29 ± 0.08	0.22 ± 0.08	0.93 ± 0.34
Gp7_unclassified	0.56 ± 0.14	0.26 ± 0.11	0.22 ± 0.05	0.57 ± 0.06	0.36 ± 0.07	0.17 ± 0.03
Peribacillus	0.38 ± 0.09	0.62 ± 0.27	0.07 ± 0.02	0.24 ± 0.06	0.24 ± 0.06	0.47 ± 0.14
Roseomonas	0.28 ± 0.06	0.25 ± 0.1	0.92 ± 0.21	0.07 ± 0.03	0.24 ± 0.06	0.09 ± 0.06
Mycobacterium	0.33 ± 0.14	0.3 ± 0.17	0.24 ± 0.05	0.27 ± 0.02	0.41 ± 0.09	0.41 ± 0.1
Stenotrophobacter	0.53 ± 0.02	0.16 ± 0.03	0.19 ± 0.1	0.29 ± 0.08	0.43 ± 0.13	0.33 ± 0.08
Terrimicrobium	0.07 ± 0.04	0.02 ± 0.02	0.1 ± 0.05	0.54 ± 0.14	0.27 ± 0.11	0.91 ± 0.13
Rubrobacter	0.44 ± 0.16	0.04 ± 0.02	0.06 ± 0.01	0.13 ± 0.06	0.49 ± 0.16	0.58 ± 0.32
Flavobacterium	0.31 ± 0.45	0.68 ± 0.98	0.13 ± 0.14	0.09 ± 0.06	0.08 ± 0.07	0.59 ± 0.73
Acidobacteria_unclassified	0.22 ± 0.07	0.18 ± 0.02	0.27 ± 0.08	0.54 ± 0.03	0.35 ± 0.05	0.4 ± 0.06
Proteobacteria_unclassified	0.17 ± 0.04	0.24 ± 0.06	0.21 ± 0.06	0.75 ± 0.1	0.16 ± 0.03	0.48 ± 0.13
Planctomycetaceae_unclassified	0.15 ± 0.03	0.21 ± 0.08	0.38 ± 0.1	0.24 ± 0.01	0.25 ± 0.03	0.56 ± 0.06
Ilumatobacter	0.09 ± 0.04	0.26 ± 0.12	0.93 ± 0.24	0.13 ± 0.01	0.15 ± 0.03	0.19 ± 0.16
Actinomarinicola	0.34 ± 0.01	0.15 ± 0.04	0.28 ± 0.11	0.47 ± 0.04	0.29 ± 0.03	0.34 ± 0.07
Rhizobiaceae_unclassified	0.07 ± 0.02	0.22 ± 0.11	0.08 ± 0.05	0.2 ± 0.2	0.76 ± 0.62	0.51 ± 0.44
Solirubrobacter	0.55 ± 0.22	0.05 ± 0.03	0.08 ± 0.05	0.44 ± 0.13	0.22 ± 0.08	0.47 ± 0.16
Syntrophorhabdus_unclassified	0.17 ± 0.09	0.67 ± 0.05	0.59 ± 0.15	0.09 ± 0.02	0.23 ± 0.1	0.04 ± 0.03
Planctomycetales_unclassified	0.22 ± 0.04	0.22 ± 0.02	0.24 ± 0.11	0.21 ± 0.03	0.25 ± 0.08	0.54 ± 0.08
Pirellula	0.14 ± 0.05	0.09 ± 0.03	0.25 ± 0.07	0.28 ± 0.04	0.27 ± 0.06	0.55 ± 0.08
Gemmata	0.29 ± 0.04	0.18 ± 0.01	0.27 ± 0.08	0.3 ± 0.06	0.25 ± 0.06	0.3 ± 0.02

Clostridiales_unclassified	0.21 ± 0.05	0.42 ± 0.06	0.34 ± 0.03	0.07 ± 0.02	0.17 ± 0.09	0.3 ± 0.29
Acidibacter_unclassified	0.3 ± 0.06	0.21 ± 0.2	0.16 ± 0.09	0.34 ± 0.04	0.24 ± 0.09	0.34 ± 0.1
Thiobacillus	0 ± 0	0.03 ± 0.02	0.19 ± 0.05	0.39 ± 0.04	0.89 ± 0.76	0.03 ± 0.02
Agromyces	0.21 ± 0.06	0.25 ± 0.06	0.1 ± 0.05	0.39 ± 0.05	0.44 ± 0.16	0.22 ± 0.14
Defluviicoccus	0.05 ± 0.02	0.01 ± 0.01	0.43 ± 0.18	0.76 ± 0.18	0.19 ± 0.08	0.18 ± 0.06
Microbacteriaceae_unclassified	0.17 ± 0.09	0.36 ± 0.08	0.16 ± 0.06	0.23 ± 0.14	0.18 ± 0.04	0.39 ± 0.3
Skermanella	0.36 ± 0.06	0.1 ± 0.04	0.09 ± 0.04	0.12 ± 0.04	0.34 ± 0.07	0.37 ± 0.2
Geminicoccaceae_unclassified	0.09 ± 0.04	0.02 ± 0.01	0.1 ± 0.02	0.35 ± 0.12	0.39 ± 0.05	0.49 ± 0.24
Planctomycetacia_unclassified	0.1 ± 0.02	0.14 ± 0.04	0.37 ± 0.07	0.21 ± 0.03	0.13 ± 0.05	0.38 ± 0.04
Gp18_unclassified	0.26 ± 0.2	0.37 ± 0.05	0.25 ± 0.06	0.2 ± 0.03	0.24 ± 0.08	0.11 ± 0.05
Acidobacteria_Gp3_unclassified	0.42 ± 0.1	0.14 ± 0.07	0.18 ± 0.02	0.33 ± 0.12	0.15 ± 0.04	0.15 ± 0.03
Litorilinea	0.22 ± 0.08	0.07 ± 0.03	0.15 ± 0.04	0.36 ± 0.01	0.38 ± 0.08	0.17 ± 0.06
Lacipirellula	0.07 ± 0.02	0.06 ± 0.02	0.14 ± 0.05	0.28 ± 0	0.15 ± 0.05	0.58 ± 0.09
Arboricoccus	0.09 ± 0.02	0.01 ± 0.01	0.3 ± 0.07	0.12 ± 0.02	0.26 ± 0.08	0.41 ± 0.1
Thermoguttaceae_unclassified	0.12 ± 0.04	0.18 ± 0.03	0.3 ± 0.1	0.18 ± 0.03	0.13 ± 0.03	0.35 ± 0.03
Cystobacteraceae_unclassified	0.33 ± 0.06	0.19 ± 0.07	0.15 ± 0.04	0.34 ± 0.17	0.23 ± 0.07	0.11 ± 0.02
Acetobacteraceae_unclassified	0.15 ± 0.03	0.05 ± 0.02	0.63 ± 0.07	0.03 ± 0.02	0.17 ± 0.03	0.11 ± 0.06
Pseudomonas	0.03 ± 0.05	0.19 ± 0.17	0.02 ± 0.01	0.11 ± 0.05	0.34 ± 0.15	0.5 ± 0.9
Blastococcus	0.17 ± 0.03	0.09 ± 0.07	0.06 ± 0.02	0.07 ± 0.01	0.29 ± 0.09	0.48 ± 0.21
Oscillochloris	0.01 ± 0.01	0 ± 0	0.83 ± 0.75	0.01 ± 0.01	0.11 ± 0.06	0.13 ± 0.07
Ignavibacterium	0.1 ± 0.05	0.19 ± 0.01	0.57 ± 0.12	0.09 ± 0.04	0.16 ± 0.06	0.05 ± 0.02
Lachnospiraceae_unclassified	0.23 ± 0.07	0.49 ± 0.16	0.07 ± 0.01	0.24 ± 0.05	0.19 ± 0.09	0.12 ± 0.07
Syntrophobacteraceae_unclassified	0.05 ± 0.02	0.02 ± 0.01	0.6 ± 0.21	0.12 ± 0.04	0.22 ± 0.05	0.08 ± 0.02
Dechloromonas	0.1 ± 0.07	0.16 ± 0.18	0.69 ± 0.36	0.03 ± 0.03	0.07 ± 0.04	0.05 ± 0.04
Desulfobulbus	0.03 ± 0.01	0.09 ± 0.03	0.6 ± 0.18	0.13 ± 0.05	0.2 ± 0.08	0.06 ± 0.03
Verrucomicrobia_unclassified	0.09 ± 0.02	0.22 ± 0.06	0.59 ± 0.18	0.05 ± 0	0.08 ± 0.03	0.06 ± 0.01
Nitrospira	0.29 ± 0.03	0.06 ± 0.05	0.08 ± 0.08	0.41 ± 0.08	0.17 ± 0.05	0.18 ± 0.14
Pedomicrobium	0.07 ± 0.03	0.08 ± 0.02	0.06 ± 0.02	0.23 ± 0.02	0.09 ± 0.03	0.52 ± 0.13
Gemmatimonadaceae_unclassified	0.47 ± 0.17	0.08 ± 0.05	0.06 ± 0.03	0.17 ± 0.01	0.22 ± 0.09	0.1 ± 0.01

Tumebacillus	0.32 ± 0.03	0.18 ± 0.03	0.15 ± 0.1	0.11 ± 0.07	0.15 ± 0.07	0.17 ± 0.11
Bacillus	0.23 ± 0.06	0.13 ± 0.02	0.18 ± 0.04	0.11 ± 0.01	0.15 ± 0.03	0.23 ± 0.08
Alsobacter	0.21 ± 0.03	0.37 ± 0.15	0.41 ± 0.04	0.02 ± 0.01	0.04 ± 0	0.02 ± 0.01
Terrimonas	0.09 ± 0.02	0.09 ± 0.05	0.13 ± 0.05	0.21 ± 0.04	0.17 ± 0.07	0.33 ± 0.11
Chryseolinea	0.02 ± 0.01	0.03 ± 0.02	0.13 ± 0.05	0.17 ± 0.05	0.17 ± 0.08	0.46 ± 0.15
Aggregatilinea	0.27 ± 0.05	0.22 ± 0.16	0.12 ± 0.03	0.16 ± 0.03	0.27 ± 0.05	0.04 ± 0
Massilia	0.12 ± 0.06	0.14 ± 0.14	0.11 ± 0.2	0.05 ± 0.02	0.11 ± 0.02	0.42 ± 0.72
Reyranella	0.21 ± 0.12	0.13 ± 0.02	0.08 ± 0.04	0.27 ± 0.04	0.21 ± 0.03	0.17 ± 0.01
Hydrogenispora	0.28 ± 0.11	0.2 ± 0.04	0.07 ± 0.04	0.09 ± 0.02	0.07 ± 0.03	0.25 ± 0.28
Methylobacter	0.16 ± 0.05	0.23 ± 0.15	0.19 ± 0.12	0.19 ± 0.08	0.25 ± 0.07	0.01 ± 0.01
Peptostreptococcaceae_unclassified	0.11 ± 0.03	0.75 ± 0.35	0.03 ± 0.01	0.03 ± 0.02	0.18 ± 0.07	0.01 ± 0.01
Gp21_unclassified	0.33 ± 0.04	0.26 ± 0.18	0.07 ± 0.06	0.15 ± 0.02	0.12 ± 0.05	0.08 ± 0.03
Thermostilla	0.09 ± 0.04	0.26 ± 0.05	0.24 ± 0.04	0.07 ± 0.04	0.13 ± 0.01	0.16 ± 0.07
Arenimonas	0.29 ± 0.17	0.19 ± 0.08	0.08 ± 0.01	0.08 ± 0.03	0.19 ± 0.05	0.12 ± 0.07
Latescibacteria_unclassified	0.18 ± 0.04	0.19 ± 0.08	0.2 ± 0.03	0.19 ± 0.03	0.14 ± 0.06	0.1 ± 0.02
Rhodospirillaceae_unclassified	0.14 ± 0.02	0.08 ± 0.02	0.1 ± 0.06	0.18 ± 0.05	0.12 ± 0.04	0.31 ± 0.07
Actinotalea	0.08 ± 0.01	0.16 ± 0.16	0.04 ± 0.02	0.08 ± 0.05	0.44 ± 0.36	0.15 ± 0.07
Erythrobacteraceae_unclassified	0.2 ± 0.15	0.15 ± 0.12	0.04 ± 0.03	0.13 ± 0.01	0.27 ± 0.06	0.13 ± 0.02
Devosia	0.07 ± 0.03	0.1 ± 0.06	0.05 ± 0.02	0.14 ± 0.05	0.27 ± 0.05	0.26 ± 0.14
Polyangiaceae_unclassified	0.1 ± 0.03	0.18 ± 0.01	0.09 ± 0.02	0.24 ± 0.1	0.18 ± 0.04	0.13 ± 0.03
Solibacillus	0.13 ± 0.04	0.53 ± 0.27	0.01 ± 0	0.04 ± 0.01	0.16 ± 0.05	0.03 ± 0.01
Syntrophaceae_unclassified	0.14 ± 0.07	0.31 ± 0.03	0.21 ± 0.05	0.1 ± 0.02	0.08 ± 0.05	0.01 ± 0.01
Candidatus_Saccharibacteria_unclassified	0.03 ± 0.02	0.15 ± 0.04	0.25 ± 0.1	0.01 ± 0.01	0.08 ± 0.05	0.19 ± 0.15
Gemmatimonadetes_unclassified	0.19 ± 0.03	0.08 ± 0.05	0.18 ± 0.04	0.14 ± 0.01	0.05 ± 0.01	0.12 ± 0.01
Porphyrobacter	0.04 ± 0.01	0.04 ± 0.02	0.3 ± 0.03	0.03 ± 0.02	0.12 ± 0.06	0.16 ± 0.08
Ramlibacter	0.22 ± 0.03	0.1 ± 0.05	0.03 ± 0.02	0.11 ± 0.05	0.24 ± 0.07	0.07 ± 0.02
Clostridiaceae_1_unclassified	0.14 ± 0.04	0.32 ± 0.18	0.09 ± 0.02	0.04 ± 0.01	0.16 ± 0.07	0.03 ± 0.03
Sporomusaceae_unclassified	0.29 ± 0.1	0.16 ± 0.04	0.05 ± 0.03	0.12 ± 0.03	0.05 ± 0.02	0.11 ± 0.08

Paludibaculum_unclassified	0.04 ± 0.02	0.1 ± 0.07	0.36 ± 0.15	0.12 ± 0.03	0.07 ± 0.01	0.05 ± 0.01
Chromatiales_unclassified	0 ± 0.01	0.02 ± 0.01	0.1 ± 0.06	0.2 ± 0	0.2 ± 0.1	0.21 ± 0.06
Saccharicenans_unclassified	0.03 ± 0.03	0.31 ± 0.16	0.25 ± 0.06	0.11 ± 0.01	0.05 ± 0.02	0.02 ± 0.02
Opitutaceae_unclassified	0.13 ± 0.01	0.05 ± 0.04	0.11 ± 0.03	0.14 ± 0.05	0.23 ± 0.16	0.09 ± 0.07
Spirochaetaceae_unclassified	0.26 ± 0.05	0.28 ± 0.18	0.12 ± 0.02	0.03 ± 0.01	0.03 ± 0.03	0.03 ± 0.03
Streptomyces	0.2 ± 0.02	0.22 ± 0.05	0.05 ± 0.01	0.12 ± 0.02	0.1 ± 0.02	0.08 ± 0.03
Flavisolibacter	0.25 ± 0.07	0.08 ± 0.05	0.01 ± 0.01	0.03 ± 0.01	0.31 ± 0.13	0.03 ± 0.03
Gp25_unclassified	0.07 ± 0.03	0.01 ± 0	0.07 ± 0.04	0.2 ± 0.02	0.08 ± 0.01	0.28 ± 0.05
Lysobacter	0.12 ± 0.03	0.14 ± 0.06	0.04 ± 0.03	0.17 ± 0.03	0.23 ± 0.07	0.03 ± 0.02
Clostridia_unclassified	0.04 ± 0.03	0.16 ± 0.02	0.18 ± 0.09	0.13 ± 0.04	0.11 ± 0.04	0.07 ± 0.03
Labilithrix	0.12 ± 0.01	0.09 ± 0.03	0.09 ± 0.02	0.16 ± 0.07	0.11 ± 0.03	0.1 ± 0.03
Sphingomonadales_unclassified	0.09 ± 0.01	0.1 ± 0.07	0.05 ± 0.03	0.12 ± 0.1	0.15 ± 0.03	0.16 ± 0.16
Paenisporosarcina	0.03 ± 0	0.05 ± 0.05	0.07 ± 0.02	0.08 ± 0.01	0.04 ± 0.01	0.33 ± 0.12
Steroidobacter	0.1 ± 0.02	0.06 ± 0.02	0.04 ± 0.02	0.2 ± 0.05	0.14 ± 0.05	0.14 ± 0.02
Oxalobacteraceae_unclassified	0.33 ± 0.16	0.1 ± 0.03	0.01 ± 0.01	0.05 ± 0.03	0.11 ± 0.04	0.03 ± 0.03
Lacipirellulaceae_unclassified	0.01 ± 0	0.01 ± 0.01	0.12 ± 0.02	0.1 ± 0.01	0.04 ± 0.02	0.27 ± 0.03
Zavarzinella	0.15 ± 0.02	0.06 ± 0.02	0.06 ± 0.03	0.11 ± 0.03	0.12 ± 0.03	0.09 ± 0.01
Phenylobacterium	0.14 ± 0.06	0.1 ± 0.06	0.04 ± 0.03	0.11 ± 0.04	0.15 ± 0.02	0.07 ± 0.03
Prosthecomicrobium	0.06 ± 0.01	0.06 ± 0.03	0.31 ± 0.02	0.01 ± 0.01	0.07 ± 0.01	0.02 ± 0.02
Selenomonadales_unclassified	0.06 ± 0.03	0.14 ± 0.07	0.22 ± 0.11	0.01 ± 0.01	0.06 ± 0.03	0.06 ± 0.02
Fulvivirgaceae_unclassified	0.03 ± 0.01	0.14 ± 0.04	0.09 ± 0.04	0.12 ± 0.02	0.09 ± 0.03	0.12 ± 0.1
Desertimonas	0.11 ± 0.02	0.07 ± 0.03	0.08 ± 0.02	0.08 ± 0.02	0.07 ± 0.01	0.14 ± 0.05
Desulfuromonas	0.11 ± 0.05	0.08 ± 0.05	0.05 ± 0.01	0.25 ± 0.18	0.08 ± 0.04	0.05 ± 0.02
Anaerolineae_unclassified	0.08 ± 0.07	0.05 ± 0.03	0.08 ± 0.02	0.1 ± 0.02	0.18 ± 0.08	0.07 ± 0.03
Paenibacillus	0.13 ± 0.03	0.17 ± 0.03	0.02 ± 0.01	0.06 ± 0.02	0.06 ± 0.02	0.12 ± 0.04
Tepidisphaera	0.13 ± 0.05	0.05 ± 0.01	0.13 ± 0.04	0.02 ± 0.01	0.05 ± 0.02	0.11 ± 0.08
Hydrogenophaga	0.06 ± 0.03	0.12 ± 0.12	0.09 ± 0.04	0.04 ± 0.03	0.14 ± 0.05	0.08 ± 0.07
Pseudonocardia	0.1 ± 0.03	0.03 ± 0.01	0.02 ± 0.01	0.08 ± 0.03	0.12 ± 0.02	0.16 ± 0.04
Syntrophobacter	0.05 ± 0.03	0.16 ± 0.03	0.2 ± 0.09	0.04 ± 0.01	0.05 ± 0.02	0.01 ± 0

Rubellimicrobium	0.12 ± 0.04	0.07 ± 0.03	0.06 ± 0.03	0.01 ± 0.01	0.17 ± 0.04	0.05 ± 0.02
Pelosinus	0.21 ± 0.23	0.08 ± 0.01	0.03 ± 0.01	0.11 ± 0.04	0.02 ± 0.01	0.07 ± 0.02
Sphingomonadaceae_unclassified	0.1 ± 0.04	0.04 ± 0.04	0.02 ± 0.02	0.09 ± 0.01	0.1 ± 0.04	0.14 ± 0.05
Fictibacillus	0.01 ± 0	0.01 ± 0.01	0.34 ± 0.03	0.01 ± 0	0.02 ± 0.01	0.03 ± 0
Chitinispirillum	0.05 ± 0.07	0.17 ± 0.1	0.06 ± 0.03	0.03 ± 0.03	0.16 ± 0.14	0.02 ± 0.01
Micromonospora	0.08 ± 0.02	0.12 ± 0.03	0.04 ± 0.02	0.05 ± 0.01	0.07 ± 0.02	0.1 ± 0.03
BRC1_unclassified	0.11 ± 0.01	0.09 ± 0.03	0.08 ± 0.02	0.04 ± 0.04	0.09 ± 0.03	0.04 ± 0.02
Desulfatiglans	0.04 ± 0.02	0.15 ± 0.02	0.08 ± 0.01	0.1 ± 0.01	0.07 ± 0.07	0.04 ± 0.03
Ferruginibacter	0.13 ± 0.02	0.04 ± 0.03	0.06 ± 0.02	0.06 ± 0.04	0.03 ± 0.01	0.1 ± 0.06
Luteitalea	0.04 ± 0.01	0.04 ± 0.01	0.08 ± 0.02	0.09 ± 0.03	0.11 ± 0.02	0.08 ± 0.05
Firmicutes_unclassified	0.09 ± 0.02	0.05 ± 0.02	0.08 ± 0.01	0.03 ± 0	0.05 ± 0.03	0.09 ± 0.08
Streptomycetaceae_unclassified	0.14 ± 0.02	0.14 ± 0.05	0.04 ± 0.01	0.04 ± 0.01	0.03 ± 0.01	0.04 ± 0.01
Turicibacter	0.06 ± 0.02	0.27 ± 0.06	0.01 ± 0	0.02 ± 0.01	0.07 ± 0.01	0.03 ± 0.03
Methylomicrobium	0.11 ± 0.04	0.01 ± 0	0.01 ± 0.01	0.13 ± 0.05	0.15 ± 0.04	0.02 ± 0.01
Fimbriiglobus	0.03 ± 0.01	0.02 ± 0	0.18 ± 0.03	0.04 ± 0.02	0.06 ± 0.02	0.05 ± 0.02
Acetivibrio	0.05 ± 0.02	0.16 ± 0.04	0.08 ± 0.04	0.02 ± 0	0.07 ± 0.02	0.04 ± 0.02
Asprobacter	0.01 ± 0.01	0.02 ± 0.02	0.09 ± 0.04	0.13 ± 0.02	0.07 ± 0.03	0.1 ± 0.02
Xanthomonadaceae_unclassified	0.1 ± 0.06	0.05 ± 0.02	0.02 ± 0.01	0.04 ± 0	0.14 ± 0.02	0.05 ± 0.03
Gp5_unclassified	0.04 ± 0.03	0.01 ± 0	0.01 ± 0.01	0.1 ± 0.03	0.04 ± 0.02	0.2 ± 0.05
Desulfomonile	0.02 ± 0	0.12 ± 0.01	0.06 ± 0.01	0.11 ± 0.03	0.08 ± 0.03	0.04 ± 0.01
Rhodanobacteraceae_unclassified	0.04 ± 0.03	0.03 ± 0.01	0.18 ± 0.04	0.03 ± 0.01	0.06 ± 0.02	0.02 ± 0.01
Parviterribacter	0.13 ± 0.03	0.04 ± 0.02	0.03 ± 0.02	0.09 ± 0.08	0.06 ± 0.04	0.04 ± 0.03
Bosea	0.01 ± 0.01	0.04 ± 0.02	0.05 ± 0.02	0.05 ± 0.03	0.1 ± 0.06	0.11 ± 0.07
Gemmatimonas	0.07 ± 0.02	0.06 ± 0.03	0.15 ± 0.08	0.01 ± 0.01	0.06 ± 0.04	0.01 ± 0.02
Psychrobacillus	0.07 ± 0.01	0.18 ± 0.07	0.01 ± 0.01	0.04 ± 0.01	0.03 ± 0.02	0.07 ± 0.04
Sporomusa	0.07 ± 0.02	0.07 ± 0.04	0.05 ± 0.02	0.06 ± 0.02	0.04 ± 0.01	0.08 ± 0.04
Ruminiclostridium	0.05 ± 0.01	0.13 ± 0.02	0.04 ± 0.01	0.06 ± 0	0.05 ± 0.02	0.04 ± 0.01
Opitutus	0.09 ± 0.03	0.08 ± 0.04	0.02 ± 0.01	0.06 ± 0	0.08 ± 0.03	0.03 ± 0.05
Longivirga	0.04 ± 0.01	0.11 ± 0.03	0.14 ± 0.05	0.02 ± 0	0.02 ± 0.01	0.02 ± 0.01

Rhodococcus	0.04 ± 0.02	0.13 ± 0.12	0.01 ± 0.01	0.02 ± 0	0.14 ± 0.03	0.02 ± 0.01
Smithella	0.03 ± 0.02	0.15 ± 0.02	0.1 ± 0.07	0.01 ± 0.01	0.05 ± 0.04	0 ± 0
Gp1_unclassified	0.2 ± 0.05	0.04 ± 0.03	0 ± 0.01	0.05 ± 0.01	0.01 ± 0.01	0.02 ± 0.01
Armatimonadetes_gp5_unclassified	0.06 ± 0.02	0.02 ± 0.02	0.03 ± 0.01	0.07 ± 0.02	0.09 ± 0.03	0.05 ± 0.02
Anaerolinea	0 ± 0	0.01 ± 0	0.13 ± 0.03	0.08 ± 0.03	0.06 ± 0.03	0.03 ± 0.01
Desulfocapsa	0 ± 0.01	0 ± 0.01	0.06 ± 0.06	0.13 ± 0.06	0.09 ± 0.05	0.05 ± 0.03
Prolixibacteraceae_unclassified	0.03 ± 0.03	0.03 ± 0.02	0.03 ± 0.01	0.01 ± 0.01	0.13 ± 0.04	0.05 ± 0.07
Clostridiales_Incertae_Sedis_XIII	0.05 ± 0.02	0.07 ± 0.03	0.07 ± 0.02	0.02 ± 0.02	0.06 ± 0.04	0.03 ± 0.02
Sandaracinus	0.07 ± 0.03	0.06 ± 0.01	0.06 ± 0.03	0.05 ± 0.02	0.05 ± 0.01	0.02 ± 0.01
Legionella	0.03 ± 0.01	0.01 ± 0	0.06 ± 0.03	0.03 ± 0.03	0.02 ± 0.01	0.12 ± 0.06
Methylophilaceae_unclassified	0.07 ± 0.06	0.11 ± 0.06	0 ± 0	0.05 ± 0.05	0.05 ± 0.03	0.05 ± 0.04
Lentimicrobium	0.04 ± 0.01	0.09 ± 0.03	0.08 ± 0.06	0.01 ± 0.01	0.06 ± 0.03	0 ± 0
Phyllobacteriaceae_unclassified	0.02 ± 0.02	0.02 ± 0.02	0.03 ± 0.01	0.04 ± 0.01	0.09 ± 0.03	0.07 ± 0.03
Ohtaekwangia	0.05 ± 0.02	0.07 ± 0.05	0.02 ± 0.03	0.06 ± 0.02	0.07 ± 0.01	0.02 ± 0.01
Rhodocyclaceae_unclassified	0.04 ± 0.02	0.04 ± 0.03	0.11 ± 0.07	0.03 ± 0.04	0.05 ± 0.03	0.01 ± 0.01
Adhaeribacter	0.14 ± 0.02	0.03 ± 0.02	0.01 ± 0.01	0.02 ± 0.01	0.05 ± 0.03	0.02 ± 0.02
Parachlamydiaceae_unclassified	0.03 ± 0.03	0 ± 0	0.04 ± 0.01	0.02 ± 0.01	0.02 ± 0.02	0.11 ± 0.06
Chloroflexales_unclassified	0.02 ± 0.01	0 ± 0	0.14 ± 0.13	0.01 ± 0	0.04 ± 0.03	0.02 ± 0.01
Geomonas	0.09 ± 0.02	0.04 ± 0.02	0.01 ± 0.01	0.06 ± 0.05	0.03 ± 0.01	0.03 ± 0.03
Geminicoccus	0.02 ± 0	0.01 ± 0.01	0.06 ± 0.01	0.02 ± 0.01	0.03 ± 0.01	0.08 ± 0.02
Gp11_unclassified	0.01 ± 0.01	0 ± 0	0.02 ± 0.01	0.07 ± 0.02	0.03 ± 0.01	0.11 ± 0.03
Kribbella	0.11 ± 0.05	0.08 ± 0.07	0 ± 0	0.01 ± 0	0.02 ± 0.01	0.01 ± 0.01
Desulfobulbaceae_unclassified	0.01 ± 0.01	0 ± 0	0.05 ± 0.01	0.08 ± 0.01	0.07 ± 0.06	0.04 ± 0.03
Altererythrobacter	0.02 ± 0.01	0.01 ± 0.01	0.01 ± 0.01	0.02 ± 0.01	0.07 ± 0.01	0.08 ± 0.02
Halieaceae_unclassified	0 ± 0	0.04 ± 0.02	0.05 ± 0.02	0.03 ± 0.02	0.04 ± 0.03	0.06 ± 0.02
Syntrophomonas	0.04 ± 0.03	0.08 ± 0.03	0.06 ± 0.02	0.01 ± 0.01	0.05 ± 0.03	0.01 ± 0.01
Piscinibacter	0.02 ± 0.02	0.01 ± 0.01	0.04 ± 0.01	0.04 ± 0.02	0.07 ± 0.03	0.04 ± 0.03
Aminicenantes_unclassified	0.01 ± 0.01	0.06 ± 0.02	0.1 ± 0.03	0.03 ± 0	0.02 ± 0.02	0 ± 0

Blastocatellaceae_unclassified	0.1 ± 0.03	0.01 ± 0.01	0.02 ± 0.02	0.01 ± 0.01	0.02 ± 0.02	0.05 ± 0.04
Kineosporiaceae_unclassified	0.04 ± 0.05	0.04 ± 0.01	0.04 ± 0.04	0.03 ± 0.01	0.06 ± 0.01	0.03 ± 0.01
Domibacillus	0.01 ± 0.01	0.01 ± 0.01	0.02 ± 0.01	0.01 ± 0.01	0.02 ± 0.01	0.13 ± 0.06
Holophagaceae_unclassified	0.04 ± 0.02	0.03 ± 0.03	0.05 ± 0.02	0.01 ± 0.01	0.02 ± 0.01	0.05 ± 0.06
Nocardioidaceae_unclassified	0.03 ± 0.01	0.02 ± 0.01	0.05 ± 0.04	0.03 ± 0.03	0.02 ± 0.01	0.05 ± 0.04
Gp22_unclassified	0.01 ± 0.01	0.04 ± 0.03	0.05 ± 0.03	0.03 ± 0.01	0.01 ± 0.01	0.06 ± 0.02
Aridibacter	0.1 ± 0.04	0.01 ± 0	0.02 ± 0.02	0.02 ± 0.01	0.04 ± 0.02	0.01 ± 0.01
Desulfuromonadaceae_unclassified	0.03 ± 0.02	0.01 ± 0.01	0 ± 0.01	0.06 ± 0.06	0.07 ± 0.03	0.02 ± 0.01
Micrococcaceae_unclassified	0.02 ± 0.01	0.09 ± 0.08	0 ± 0	0.01 ± 0.01	0.07 ± 0.02	0.01 ± 0.01
Sphaerobacteraceae_unclassified	0.09 ± 0.02	0.01 ± 0.01	0 ± 0.01	0.04 ± 0.01	0.04 ± 0.01	0.01 ± 0
Actinomycetospora	0.08 ± 0.02	0.04 ± 0.01	0.02 ± 0.01	0.01 ± 0.01	0.02 ± 0.01	0.02 ± 0.01
Sporacetigenium	0.05 ± 0.02	0.02 ± 0.01	0 ± 0	0.04 ± 0.01	0.04 ± 0.01	0.04 ± 0.01
Microlunatus	0.01 ± 0.01	0.01 ± 0	0.01 ± 0	0.02 ± 0.01	0.04 ± 0.02	0.08 ± 0.03
Gracilibacter	0.05 ± 0.01	0.08 ± 0.02	0.01 ± 0.01	0.02 ± 0.02	0.01 ± 0.01	0.03 ± 0.01
Thermoanaerobaculum	0.02 ± 0.01	0.03 ± 0.01	0.08 ± 0.05	0.02 ± 0	0.02 ± 0.02	0 ± 0
Syntrophus	0.02 ± 0.01	0.07 ± 0.03	0.03 ± 0.02	0 ± 0	0.06 ± 0.04	0 ± 0
Polyangium	0.03 ± 0.01	0.01 ± 0.01	0.02 ± 0	0.06 ± 0.01	0.06 ± 0.02	0.01 ± 0.01
Neobacillus	0.04 ± 0.01	0.03 ± 0.02	0.01 ± 0.01	0.03 ± 0.01	0.02 ± 0	0.05 ± 0.02
Methylobacterium	0.01 ± 0	0.01 ± 0.01	0.02 ± 0.01	0.01 ± 0.02	0.01 ± 0.01	0.09 ± 0.1
Cytobacillus	0.03 ± 0.02	0.03 ± 0.01	0.05 ± 0.01	0.02 ± 0	0.02 ± 0.01	0.02 ± 0.01
Mesorhizobium	0.01 ± 0	0 ± 0	0.01 ± 0	0.03 ± 0.02	0.02 ± 0.02	0.08 ± 0.04
Flavitalea	0.09 ± 0.02	0.01 ± 0.01	0.01 ± 0.01	0.02 ± 0.01	0.02 ± 0.01	0.02 ± 0.01
Virgisporangium	0.02 ± 0.01	0.03 ± 0.02	0.03 ± 0.05	0.02 ± 0.02	0.04 ± 0	0.03 ± 0.02
Polaromonas	0.06 ± 0.04	0.04 ± 0.01	0.02 ± 0.04	0.01 ± 0.01	0.02 ± 0.02	0 ± 0
Caulobacter	0.02 ± 0.01	0.02 ± 0.02	0.01 ± 0.01	0.03 ± 0.03	0.08 ± 0.05	0.01 ± 0.01
Saprospirales_unclassified	0.04 ± 0.02	0.03 ± 0.02	0.03 ± 0.01	0.02 ± 0	0.02 ± 0.01	0.01 ± 0.01
Methyloceanibacter	0.05 ± 0.04	0.01 ± 0.01	0.01 ± 0.01	0.04 ± 0.02	0.01 ± 0.01	0.04 ± 0.01
Beijerinckia	0.06 ± 0.02	0.05 ± 0.02	0.01 ± 0	0.01 ± 0.01	0.02 ± 0.01	0.01 ± 0
Ureibacillus	0.01 ± 0.01	0.06 ± 0.01	0.01 ± 0.01	0.01 ± 0	0.03 ± 0.01	0.02 ± 0.01

Nitrosomonadales_unclassified	0.02 ± 0.01	0.02 ± 0.01	0 ± 0	0.01 ± 0	0.01 ± 0.01	0.07 ± 0.03
Lacibacter	0.02 ± 0.01	0.02 ± 0	0 ± 0.01	0.03 ± 0.02	0.04 ± 0.01	0.03 ± 0.02
Iamia	0.01 ± 0.01	0 ± 0	0.04 ± 0.02	0.02 ± 0.01	0.03 ± 0.01	0.02 ± 0.01
Planctopirus	0.01 ± 0	0.01 ± 0.01	0.05 ± 0.01	0.03 ± 0.01	0 ± 0	0.03 ± 0.01
Aetherobacter	0.01 ± 0.01	0.07 ± 0.01	0.01 ± 0.01	0.01 ± 0	0.03 ± 0.02	0.01 ± 0
Povalibacter	0.01 ± 0.01	0.03 ± 0.03	0 ± 0.01	0.06 ± 0.01	0.01 ± 0.01	0.03 ± 0.01
Anaerobacterium	0.04 ± 0.01	0.05 ± 0.03	0.03 ± 0.02	0.01 ± 0.01	0.01 ± 0.01	0 ± 0
Myxococcus	0.02 ± 0.02	0.02 ± 0.01	0.03 ± 0.01	0.03 ± 0.03	0.02 ± 0.01	0.01 ± 0
Paenibacillaceae_1_unclassified	0.03 ± 0.01	0.04 ± 0.02	0.01 ± 0.01	0.02 ± 0.01	0.01 ± 0.01	0.02 ± 0
Pseudolabrys	0.03 ± 0.03	0.05 ± 0.01	0 ± 0	0.01 ± 0.01	0.01 ± 0.01	0.02 ± 0.01
Dactylosporangium	0.02 ± 0.01	0.03 ± 0.01	0 ± 0	0.02 ± 0.02	0.02 ± 0.01	0.03 ± 0.01
Sporobacter	0.05 ± 0.07	0.03 ± 0.02	0.01 ± 0.01	0.01 ± 0	0.01 ± 0	0.01 ± 0.01
Propionivibrio	0.02 ± 0	0.03 ± 0.04	0.03 ± 0.02	0.01 ± 0.01	0.02 ± 0.01	0.01 ± 0.01
Nannocystis	0.01 ± 0.01	0.01 ± 0	0.03 ± 0.01	0.02 ± 0	0.04 ± 0.02	0 ± 0.01
Nonomuraea	0.04 ± 0.01	0.03 ± 0.01	0 ± 0	0 ± 0	0.02 ± 0.01	0.01 ± 0
Dongia	0.01 ± 0.01	0.01 ± 0.01	0.01 ± 0	0.02 ± 0	0.04 ± 0.01	0.01 ± 0.01
Streptosporangiaceae_unclassified	0.04 ± 0.03	0.02 ± 0.01	0 ± 0	0 ± 0	0.01 ± 0	0.01 ± 0
Caulobacteraceae_unclassified	0.02 ± 0.01	0.01 ± 0	0 ± 0	0.02 ± 0.01	0.03 ± 0.01	0.01 ± 0
Dehalococcoidaceae_unclassified	0.02 ± 0.01	0.01 ± 0.01	0.01 ± 0	0.03 ± 0.01	0.01 ± 0.01	0.01 ± 0

Table S5: Relative abundance of protist core communities in each sampling site (mean ± standard deviation).

	Pond			Spring		
**Taxon**	**Bison**	**Cow**	**Non-grazing**	**Bison**	**Cow**	**Non-grazing**
Acanthamoeba	0.33 ± 0.28	0.49 ± 0.42	0.14 ± 0.05	0.55 ± 0.12	0.45 ± 0.32	0.22 ± 0.09
Actinocephalidae_unclassified	0.11 ± 0.1	0.45 ± 0.37	1.34 ± 0.44	1.77 ± 1.16	0.8 ± 0.45	9.85 ± 5.82
Allantion	0.06 ± 0.06	0.12 ± 0.09	0.1 ± 0.07	0.07 ± 0.04	0.03 ± 0.02	0.11 ± 0.09
Allapsidae_unclassified	0.59 ± 0.23	0.94 ± 0.14	0.88 ± 0.31	0.78 ± 0.32	0.7 ± 0.31	1.19 ± 0.61
Allapsidae	0.37 ± 0.01	0.2 ± 0.09	0.1 ± 0.08	0.58 ± 0.48	0.19 ± 0.1	0.23 ± 0.07
AND16.lineage	1.04 ± 1.03	0.48 ± 0.52	0.04 ± 0.05	0.33 ± 0.24	0.3 ± 0.03	0.15 ± 0.1
Aphamonas	0.19 ± 0.1	0.22 ± 0.18	0.15 ± 0.06	0.11 ± 0.03	0.14 ± 0.06	0 ± 0
Aphanomyces	0.18 ± 0.16	0.62 ± 0.99	0.06 ± 0.06	0.29 ± 0.3	0.04 ± 0.05	0.02 ± 0.03
Apicomplexa_unclassified	0.18 ± 0.35	0.14 ± 0.14	0.05 ± 0.05	0.13 ± 0.13	0.73 ± 0.35	0.96 ± 0.3
Breviata.lineage_unclassified	0.05 ± 0.01	0.31 ± 0.11	0.06 ± 0.04	1.01 ± 1.19	0.39 ± 0.16	0.02 ± 0.02
Breviata.lineage	0.09 ± 0.04	0.06 ± 0.07	0.05 ± 0.03	0.31 ± 0.34	0.14 ± 0.05	0.05 ± 0.04
Cercomonadida_unclassified	0.06 ± 0.03	0.12 ± 0.06	0.1 ± 0.12	0.26 ± 0.11	0.06 ± 0.05	0.13 ± 0.03
Cercomonadidae_unclassified	0.1 ± 0.05	0.03 ± 0.03	0.12 ± 0.09	0.38 ± 0.08	0.18 ± 0.04	0.39 ± 0.17
Cercomonas	0.65 ± 0.26	0.6 ± 0.33	0.62 ± 0.16	1.42 ± 0.14	0.53 ± 0.3	1.17 ± 0.72
Cercozoa_unclassified	3.84 ± 1	5.48 ± 3.59	3.61 ± 0.75	4.68 ± 1.99	3.54 ± 1.27	3 ± 0.56
Cercozoa	0.28 ± 0.09	0.24 ± 0.22	0.32 ± 0.04	0.15 ± 0.05	0.13 ± 0.07	0.4 ± 0.22
Chilodonellidae	0.06 ± 0.07	0.23 ± 0.21	0.15 ± 0.15	0.13 ± 0.07	0.38 ± 0.25	0.09 ± 0.06
Chlamydomonadales_unclassified	3.21 ± 1.35	2.46 ± 1.98	0.92 ± 0.19	0.79 ± 0.66	0.78 ± 0.58	0.13 ± 0.04
Chlamydomyzium	0.09 ± 0.07	0.36 ± 0.34	0.06 ± 0.09	0.02 ± 0.03	0.32 ± 0.43	0.17 ± 0.24
Chlorellales_unclassified	0.62 ± 0.23	0.27 ± 0.06	0.67 ± 0.09	0.28 ± 0.16	0.3 ± 0.15	0.1 ± 0.02
Chloromonas	0.05 ± 0.04	0.03 ± 0.04	0.21 ± 0.05	0.01 ± 0.01	0.03 ± 0.03	0.1 ± 0.11
Chlorophyceae_unclassified	0.39 ± 0.11	0.36 ± 0.04	1.36 ± 0.37	0.15 ± 0.19	0.23 ± 0.28	0.01 ± 0.03
Chrysophyceae_Clade.C_unclassified	0.28 ± 0.19	0.12 ± 0.14	0.17 ± 0.1	0.13 ± 0.04	0.15 ± 0.11	0.06 ± 0.04
Chrysophyceae_unclassified	0.11 ± 0.1	0.17 ± 0.11	0.27 ± 0.16	0.06 ± 0.05	0.19 ± 0.14	0.01 ± 0.02
Colpodida_unclassified	0.32 ± 0.11	0.23 ± 0.13	0.39 ± 0.3	0.92 ± 0.56	3.62 ± 1.84	1.16 ± 0.38

Conosa_unclassified	0.02 ± 0.01	0.27 ± 0.25	0.06 ± 0.05	0.22 ± 0.16	0.04 ± 0.03	0.17 ± 0.11
Craticula	0.8 ± 0.55	0.74 ± 0.29	0.34 ± 0.18	0.02 ± 0.01	0.27 ± 0.29	0.01 ± 0.01
Cyrtolophosidida_unclassified	0.14 ± 0.11	0.13 ± 0.1	0.07 ± 0.04	0.3 ± 0.34	0.24 ± 0.09	0.09 ± 0.04
Echinamoeba	0.48 ± 0.51	0.42 ± 0.35	0.06 ± 0.03	0.69 ± 0.15	0.79 ± 0.28	0.08 ± 0.02
Eocercomonas	0.56 ± 0.23	0.44 ± 0.16	0.54 ± 0.35	1.49 ± 0.26	0.68 ± 0.14	1.36 ± 0.32
Euglyphida_unclassified	0.29 ± 0.13	0.41 ± 0.33	0.12 ± 0.15	0.68 ± 0.3	0.37 ± 0.11	0.28 ± 0.07
Euglyphida	0.1 ± 0.1	0.01 ± 0.01	0.1 ± 0.03	0.03 ± 0.03	0.05 ± 0.03	0.13 ± 0.08
Eustigmatophyceae_unclassified	2.15 ± 0.84	1.37 ± 0.41	1.62 ± 0.33	0.09 ± 0.14	0.02 ± 0.03	0.13 ± 0.04
Filosa.Sarcomonadea_unclassified	0.13 ± 0.05	0.57 ± 0.28	0.02 ± 0.01	0.14 ± 0.08	0.83 ± 0.9	0.16 ± 0.12
Filosa.Sarcomonadea	0.41 ± 0.22	1.11 ± 1.01	0.04 ± 0.04	0.33 ± 0.11	1.19 ± 1.05	0 ± 0
Flamella.lineage_unclassified	0.1 ± 0.03	0.32 ± 0.1	0.04 ± 0.02	0.15 ± 0.04	0.23 ± 0.13	0.05 ± 0.06
Flectomonas	0.15 ± 0.08	0.15 ± 0.07	0.04 ± 0.03	0.14 ± 0.03	0.09 ± 0.06	0.08 ± 0.03
Glissomonadida_Clade.Y	0.61 ± 0.48	0.1 ± 0.05	0.23 ± 0.03	0.28 ± 0.1	0.1 ± 0.05	0.9 ± 0.72
Glissomonadida_unclassified	0.25 ± 0.08	0.43 ± 0.32	0.14 ± 0.09	0.32 ± 0.11	0.37 ± 0.18	0.69 ± 0.31
Glissomonadida	2.78 ± 1.82	0.74 ± 0.72	0.21 ± 0.17	0.64 ± 0.21	0.6 ± 0.35	0.4 ± 0.25
Gomphonema	0.17 ± 0.1	0.22 ± 0.24	0.4 ± 0.41	0 ± 0.01	0.09 ± 0.08	0.69 ± 0.5
Gregarines	0.26 ± 0.07	0.34 ± 0.12	0.23 ± 0.07	0.36 ± 0.05	0.36 ± 0.16	0.28 ± 0.08
Group.Te	0.51 ± 0.27	0.54 ± 0.16	0.46 ± 0.25	0.52 ± 0.14	0.46 ± 0.12	0.93 ± 0.52
Hartmannella	0.72 ± 0.2	0.48 ± 0.44	0.27 ± 0.2	0.95 ± 0.41	1.1 ± 0.06	0.26 ± 0.13
Hartmannellidae_unclassified	0.03 ± 0.03	0.12 ± 0.11	0 ± 0.01	0.8 ± 0.69	0.25 ± 0.1	0.13 ± 0.09
Hyphochrytriaceae_unclassified	0.17 ± 0.07	0.33 ± 0.15	0.11 ± 0.1	0.35 ± 0.12	0.23 ± 0.2	0.09 ± 0.03
Hyphochytrium	0.71 ± 0.36	1.98 ± 3.12	0.35 ± 0.34	0.42 ± 0.13	0.28 ± 0.14	0.3 ± 0.17
Hypotrichia_unclassified	1.65 ± 0.8	2.44 ± 1.79	1.84 ± 0.19	3.12 ± 0.48	4.01 ± 0.94	0.45 ± 0.11
Limnofilidae_unclassified	0.06 ± 0.03	0.03 ± 0.02	0.06 ± 0.04	0.05 ± 0.02	0.05 ± 0.03	0.11 ± 0.04
Limnofilidae	0.11 ± 0.02	0.02 ± 0.02	0.04 ± 0.04	0.23 ± 0.09	0.09 ± 0.02	0.09 ± 0.05
Litostomatea_unclassified	0.62 ± 0.28	0.5 ± 0.39	0.51 ± 0.35	0.37 ± 0.26	0.87 ± 0.66	0.21 ± 0.17
Litostomatea	0.69 ± 0.65	0.3 ± 0.23	0.16 ± 0.21	0.14 ± 0.15	0.22 ± 0.11	0.25 ± 0.19
LKM74.lineage	0.07 ± 0.03	0.06 ± 0.06	0.11 ± 0.05	0.09 ± 0.02	0.05 ± 0.04	0.13 ± 0.04
MAST.12C	0.14 ± 0.07	0.08 ± 0.07	0.04 ± 0.03	0.16 ± 0.02	0.08 ± 0.07	0.01 ± 0.02

Mb5C.lineage	0.85 ± 0.3	0.52 ± 0.2	0.96 ± 0.34	0.65 ± 0.17	0.37 ± 0.22	0.82 ± 0.31
Metabolomonas	0.05 ± 0.02	0.11 ± 0.08	0.08 ± 0.02	0.14 ± 0.08	0.07 ± 0.01	0.11 ± 0.13
Monocystis	0.05 ± 0.07	1.36 ± 0.55	6.11 ± 0.72	7.21 ± 2.04	8.48 ± 4.29	3.6 ± 2.58
Monosigidae_Group_O	0.03 ± 0.01	0 ± 0.01	0.01 ± 0.02	0.11 ± 0.02	0.02 ± 0.02	0.25 ± 0.29
Navicula	8.37 ± 10.41	6.09 ± 8.35	1.14 ± 1.15	0.63 ± 0.52	1.64 ± 0.99	0.43 ± 0.47
NC12A.lineage	0.09 ± 0.06	0.08 ± 0.07	0.06 ± 0.03	0.12 ± 0.06	0.04 ± 0.02	0.04 ± 0.07
NC12B.lineage	1.17 ± 0.36	1.52 ± 0.22	0.6 ± 0.18	0.97 ± 0.52	0.83 ± 0.44	0.16 ± 0.16
Neochlorosarcina	0.41 ± 0.14	0.2 ± 0.17	0.05 ± 0.06	0.15 ± 0.11	0.29 ± 0.3	0.06 ± 0.04
Neoheteromita	0.22 ± 0.04	0.47 ± 0.33	0.28 ± 0.18	0.61 ± 0.11	0.22 ± 0.09	1.63 ± 0.53
Nitzschia	0.96 ± 0.71	0.67 ± 0.17	0.15 ± 0.06	0.06 ± 0.06	1.52 ± 1.54	0.32 ± 0.3
Nolandellidae	1.09 ± 0.37	1.08 ± 0.59	0.38 ± 0.2	2.82 ± 1.54	1.58 ± 0.53	0.57 ± 0.21
Novel.Gran.234	0.08 ± 0.04	0.04 ± 0.03	0.02 ± 0.02	0.04 ± 0.02	0.03 ± 0.02	0.07 ± 0.02
Novel.Gran.5	0.09 ± 0.05	0.05 ± 0.04	0.09 ± 0.06	0.07 ± 0.07	0.08 ± 0.02	0.26 ± 0.1
Novel.Gran.6	0.09 ± 0.05	0.09 ± 0.02	0.13 ± 0.06	0.18 ± 0.04	0.05 ± 0.04	0.03 ± 0.04
Ochromonas	0.01 ± 0.01	0.13 ± 0.06	0.94 ± 0.61	1.02 ± 0.85	0.15 ± 0.07	0.3 ± 0.3
Ochrophyta_unclassified	0.06 ± 0.03	0.26 ± 0.08	0.17 ± 0.06	0.07 ± 0.1	0.05 ± 0.06	0.13 ± 0.15
Oedogonium	0.78 ± 0.32	0.24 ± 0.16	0.99 ± 0.35	0.28 ± 0.22	0.06 ± 0.09	0 ± 0
Oomycota_unclassified	0.32 ± 0.12	0.83 ± 0.38	1.07 ± 1.36	0.78 ± 0.2	0.96 ± 0.74	3.72 ± 4.14
Oxytrichidae_unclassified	0.37 ± 0.38	0.14 ± 0.2	0.14 ± 0.07	0.15 ± 0.13	0.42 ± 0.29	0.12 ± 0.12
Paracercomonas	1.32 ± 0.65	1.5 ± 1.27	1.3 ± 0.79	2.37 ± 0.48	1.54 ± 0.87	1.63 ± 0.66
Paulinella	0 ± 0	0.47 ± 0.35	0.18 ± 0.13	0.18 ± 0.09	0.2 ± 0.05	0.05 ± 0.03
Peronosporales_unclassified	1.34 ± 0.34	4.65 ± 3.07	1.03 ± 0.21	3.76 ± 2.3	3.89 ± 0.9	1.92 ± 1.89
Peronosporales	0.04 ± 0.01	0.14 ± 0.11	0.07 ± 0.02	0.13 ± 0.08	0.03 ± 0.02	0.14 ± 0.16
Pinnularia	0.18 ± 0.18	1.72 ± 1.24	2.71 ± 3.73	0.99 ± 0.56	9.87 ± 11.31	1.25 ± 1.24
Pseudodendromonadales_unclassified	0.25 ± 0.1	0.32 ± 0.04	0.07 ± 0.02	1.05 ± 0.32	0.3 ± 0.09	0.08 ± 0.06
Pseudodifflugiidae	0.71 ± 0.05	1.56 ± 1.04	0.45 ± 0.07	0.24 ± 0.14	0.63 ± 0.7	0.23 ± 0.16
Pseudoperkinsidae_unclassified	0.2 ± 0.09	0.07 ± 0.04	0.03 ± 0.02	0.04 ± 0.02	0.04 ± 0.04	0.16 ± 0.09
Pseudoperkinsidae	0.17 ± 0.06	0.23 ± 0.15	0.33 ± 0.11	0.01 ± 0.01	0.01 ± 0.01	0.35 ± 0.44

Pythium	1.03 ± 0.22	1.55 ± 1.06	0.71 ± 0.64	1.61 ± 0.67	1.64 ± 1.64	1.08 ± 0.44
Raphid.pennate_unclassified	0.65 ± 0.19	0.25 ± 0.15	0.06 ± 0.07	0.29 ± 0.18	1.06 ± 0.74	0.49 ± 0.44
Rhogostoma	0.14 ± 0.12	0.07 ± 0.09	0.04 ± 0.04	0.44 ± 0.71	0.26 ± 0.3	3.43 ± 3.2
Rhogostoma.lineage_unclassified	0.07 ± 0.06	0.93 ± 0.19	0.03 ± 0.04	0.57 ± 0.42	0.2 ± 0.12	0.18 ± 0.12
Rhogostoma.lineage	2.17 ± 0.64	2.7 ± 0.85	1.48 ± 0.81	3.09 ± 0.76	4.06 ± 1.2	2.65 ± 2.04
Sandona	0.9 ± 0.27	1.32 ± 0.27	0.57 ± 0.27	1.99 ± 1.77	0.74 ± 0.39	1.03 ± 0.32
Sandonidae_Clade.N.F.A	0.05 ± 0.02	0.07 ± 0.05	0.03 ± 0.01	0.13 ± 0.09	0.02 ± 0.04	0.03 ± 0.02
Sandonidae_unclassified	7.17 ± 2.42	6.01 ± 0.86	7.44 ± 6.38	6.67 ± 2.65	4.03 ± 1.66	3.83 ± 1.15
Sandonidae	1.35 ± 0.6	0.98 ± 0.77	0.48 ± 0.23	2.37 ± 0.61	0.68 ± 0.27	0.79 ± 0.26
Schizoplasmodiids_unclassified	0.04 ± 0.03	0.05 ± 0.05	0.12 ± 0.03	0.16 ± 0.1	0.09 ± 0.1	0.28 ± 0.14
Sellaphora	0.09 ± 0.09	1.29 ± 0.26	0.11 ± 0.04	0.2 ± 0.06	0.02 ± 0.02	0.12 ± 0.09
Sphaeropleales_unclassified	5.03 ± 1.65	1.81 ± 0.41	7.24 ± 0.66	0.11 ± 0.04	0.75 ± 0.33	0.09 ± 0.06
Spongospora	0.18 ± 0.16	0.21 ± 0.09	0.21 ± 0.18	0.04 ± 0.04	0.1 ± 0.11	0.05 ± 0.06
Spumella	0.3 ± 0.08	0.35 ± 0.18	0.13 ± 0.09	0.43 ± 0.08	0.32 ± 0.25	0.29 ± 0.12
Teretomonas	0.16 ± 0.07	0.19 ± 0.01	0.13 ± 0.06	0.11 ± 0.07	0.1 ± 0.05	0.23 ± 0.05
Thaumatomonas	0.06 ± 0.05	0 ± 0.01	0.01 ± 0.01	0.2 ± 0.17	0.1 ± 0.06	0.33 ± 0.09
Thraustochytriaceae_unclassified	0.39 ± 0.15	0.42 ± 0.22	0.12 ± 0.03	0.75 ± 0.22	0.72 ± 0.38	0.68 ± 0.42
Thraustochytriaceae	0.05 ± 0.03	0.13 ± 0.1	0.04 ± 0.03	0.2 ± 0.07	0.1 ± 0.07	0.07 ± 0.03
Tribonema	0.02 ± 0.02	0.03 ± 0.01	0.04 ± 0.04	0.05 ± 0.03	0.19 ± 0.18	0 ± 0
Trimastix	0.16 ± 0.07	0.16 ± 0.11	0.08 ± 0.04	0.38 ± 0.43	0.19 ± 0.15	0.05 ± 0.07
Trinematidae	0.23 ± 0.18	0.01 ± 0.01	0.18 ± 0.1	0.18 ± 0.04	0.08 ± 0.06	0.4 ± 0.08
Tubulinea_unclassified	0.68 ± 0.27	1.3 ± 0.25	0.28 ± 0.23	0.64 ± 0.26	0.92 ± 0.47	0.29 ± 0.17
Variosea_unclassified	0.14 ± 0.05	0.15 ± 0.09	0.02 ± 0.02	0.33 ± 0.18	0.15 ± 0.07	0.23 ± 0.24
Variosea	0.03 ± 0.03	0.02 ± 0.02	0.01 ± 0.02	0.04 ± 0.04	0.03 ± 0.02	0.14 ± 0.03
Viridiraptor	0.02 ± 0.02	0.05 ± 0.03	0.06 ± 0.01	0.02 ± 0.02	0.02 ± 0.01	0.06 ± 0.06
Xanthophyceae_unclassified	0.05 ± 0.03	0.06 ± 0.05	0.25 ± 0.09	0.19 ± 0.09	0.13 ± 0.07	0.02 ± 0.01
